# “Turn-On” Fluorescent Biosensors for High Selective and Sensitive Detection of Al^3+^ Ion

**DOI:** 10.3389/fchem.2020.607614

**Published:** 2020-11-19

**Authors:** Pengfei Wang, Lijie Liu, Fanda Meng, Maroof Ahmad Khan, Hui Li

**Affiliations:** ^1^Key Laboratory of Cluster Science of Ministry of Education, School of Chemistry and Chemical Engineering, Beijing Institute of Technology, Beijing, China; ^2^Institute of Basic Medicine, Shandong First Medical University & Shandong Academy of Medical Sciences, Shandong, China

**Keywords:** pyrrole hydrazone Schiff base, fluorescent biosensors, detection of Al^3+^, theoretical calculation, test paper

## Abstract

A series of new compounds (**1**-**4**) based on pyrrole hydrazone Schiff bases were designed and synthesized. The interactions of these new compounds with metal ions and their fluorescent recognition were investigated. All compounds showed “turn-on” fluorescence in the presence of Al^3+^ in aqueous solution. Their sensing behaviors with Al^3+^ were studied using photophysical experiments, ESI-MS spectrometry analysis, ^1^H NMR titration, and DFT calculation. The detection limits of **1**-**4** for the analysis of Al^3+^ were found to reach a 10^−8^ M level in aqueous solution, which are far lower than the WHO guidelines for drinking water (7.41 mM for Al^3+^). A high selectivity test paper has been fabricated for Al^3+^ detection based on sensor **3**. Theoretical calculations (DFT) have been carried out to elucidate the configuration of **1**-**4** and their Al complexes and rationalize experimental absorption data.

## Introduction

Aluminum is widely used as a common metal, the content is second only to oxygen and silicon, ranking third, and is the most abundant metal element in the Earth's crust (Das et al., 2013; Sahana et al., 2013). Aluminum and its compounds have been widely used in food additives (Aguilar et al., 2008; Kim et al., 2012), water treatment, pharmaceuticals, and light alloy production (DeVoto and Yokel, 1994; Greger et al., 1997; Berthon, 2002). The toxic effects of Al^3+^ ions not only affect plants and aquatic ecosystems, but they also affect humans. Long-term exposure to aluminum can cause harm to human bodies and organs including the onset of diseases such as Alzheimer's disease and Parkinson's disease, and memory loss and cardiac arrest (Liao et al., 2013; Chatterjee et al., 2016; Kumawat et al., 2016; Liu et al., 2016) or even threaten life. Aluminum products were listed in the first category of carcinogens as a preliminary list of carcinogens. According to the World Health Organization's assessment, the daily intake of aluminum is specified to be 3–10 mg/day, and the limits of Al^3+^ concentration in drinking water is 7.41 mM (Valeur and Leray, 2000; Barcelo and Poschenrieder, 2002; Krejpcio and Wojciak, 2002). Fluorescence sensors have generated extensive research in the past few decades, due to their simple operation (Kong et al., 2014; Singh et al., 2017; Sharma et al., 2019; Shi et al., 2020), extremely high sensitivity (Dong et al., 2017; Fu J. et al., 2019; Li et al., 2020), and direct visual effect (Fu W. et al., 2019; Tang et al., 2019; Jiao et al., 2020) and high selectivity (Li X. et al., 2014; Jiang et al., 2017). Most Al^3+^ sensors are designed with quite complex structures, which are difficult to synthesize and dissolve in water (Li et al., 2013; Li T. et al., 2014; Gupta and Kumar, 2016; Huang et al., 2016; Balakrishnan et al., 2017). Therefore, it is necessary to develop a water-soluble Al^3+^ sensor that can be easily synthesized and can detect Al^3+^ with high selectivity in aqueous environments even at very low concentrations.

**Scheme 1:**
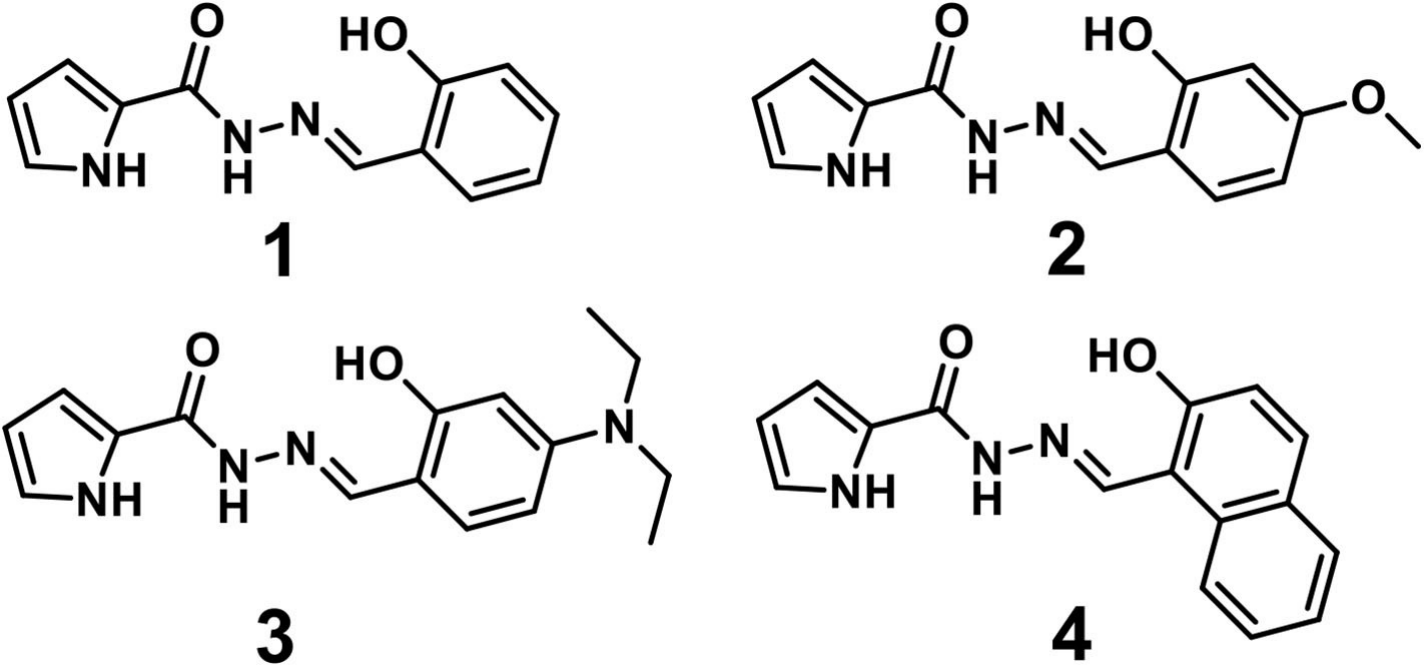
The illustration of molecular structures of sensors **1**-**4**.

The hydrazone Schiff base has been widely investigated for convenient synthesis, adjustable electronic properties, and excellent chelating ability (Boonkitpatarakul et al., 2015). The thiophene- and furan-hydrazide hydrazone Schiff bases have been used as fluorescent sensors for metal ions, anions, and organic acids (Boonkitpatarakul et al., 2015; Hwang et al., 2017; Jeong et al., 2017). In addition, diethylaminophenol has also been selected as an ideal component of many chemical sensors because it is a well-known chromophore that has hydrophilic properties and functions (Aguilar et al., 2008; Jeong et al., 2017). However, pyrrole hydrazone Schiff bases, as a common heterocyclic compound are rarely studied. A pyrrole hydrazone Schiff base also has potential application value in ion recognition owing to its hydrazone structure. Our group has reported some research results in the field of fluorescence detection in the early stage (Hao et al., 2016; Song et al., 2020). In this work, we reported a series of chemosensors based on pyrrole hydrazone Schiff base derivatives (Scheme 1). Sensor **1** was synthesized referring to a literature method (Hanna et al., 2007). Our study found that it had a high selectivity Al^3+^ fluorescence recognition property. We look forward to designing and changing its structure with **1** as the core structure, so that the emission spectrum can be shifted to longer wavelengths, but its high-efficiency ion recognition properties are not affected. We designed and synthesized sensors **2** and **3** as the electron-donating substituents N, N-diethyl and methoxy groups were introduced to the benzene ring, which were beneficial to improve HOMO level of molecules. The strategy of extensive π-conjugation was applied in sensor **4** by replacing the benzene ring with a naphthalene ring. We expect that the designed sensors 2-4 have more efficient and selective fluorescence recognition properties for Al^3+^ and have more responsive long-wave colors in the emission spectrum.

## Experimental Section

### Materials

All solvents and reagents (analytical and spectral) were obtained from Sigma-Aldrich and used as standard.

### Measurements and Instruments

^1^H NMR and ^13^C NMR measurements were performed on a Bruker AVANCE III HD 400 MHz nuclear magnetic resonance spectrometer, the ^1^H-^1^H COSY NMR spectrum were performed on a Bruker AVANCE III HD 700 MHz nuclear magnetic resonance spectrometer. Electrospray ionization mass spectrometry (ESI-MS) was collected on an AGILENT Q-TOF 6520 LC-MS instrument. Absorption spectra were collected at room temperature using a PERSEE TU-1950 double-beam UV-Vis spectrophotometer. The fluorescence emission spectrum was collected on a HITACHI F-7000 fluorescence spectrophotometer. Absolute PL quantum yield was collected using a Quantaurus-QY Absolute PL quantum yield spectrometer C11347-11 | Hamamatsu Photonics. IR spectrums were collected using a Thermo IS5 Fourier transform infrared spectrometer. Elemental analysis of carbon, nitrogen, and hydrogen was performed using an EA3000 elemental analyzer at the Analysis and Testing Center of Beijing Institute of Technology.

### Preparation of Compounds

#### 1H-Pyrrole-2-carbohydrazide

N_2_H_4_.H_2_O (80% in water, 30 ml) and 1H-pyrrole-2-carboxylic acid ethyl ester (10 g) was added to a 100 ml Schlenk flask. After vacuumizing and filling with nitrogen three times, the reaction was placed in a 70°C oil bath and kept for 12 h (Pawel et al., 2009). After the reaction, the resulting reaction suspension was filtered and washed with cold water to obtain 8.5 g (94%) of the product as colorless crystals, ^1^H NMR (400 MHz, DMSO-*d*_6_) δ 11.42 (s, 1H), 9.22 (s, 1H), 6.83 (td, *J* = 2.7, 1.4 Hz, 1H), 6.73 (ddd, *J* = 3.9, 2.5, 1.5 Hz, 1H), 6.05 (dt, *J* = 3.7, 2.4 Hz, 1H), 4.28 (s, 2H).

A typical synthesis of sensors **1**-**4** was performed by refluxing a mixture of o-hydroxybenzaldehyde (1.0 mmol) and 1*H*-pyrrole-2-carbohydrazide (1.01 mmol) in 10 mL of methanol for 24 h. The resulting mixture was cooled down to room temperature. The precipitate was filtered off and washed with cold methanol and cold water. The resulting solid products were obtained by characterization by ^1^H NMR, ^13^C NMR, and HRMS.

#### (*E*)-N′-(2-hydroxybenzylidene)-1H-pyrrole-2-carbohydrazide (1)

The precipitate was collected in 96% yield as white powder. ^1^H NMR (400 MHz, DMSO-*d*_6_) δ 11.76 (s, 1H), 11.71 (s, 1H), 11.31 (s, 1H), 8.53 (s, 1H), 7.64–7.46 (m, 1H), 7.28 (t, *J* = 6.9 Hz, 1H), 7.00 (d, *J* = 1.4 Hz, 2H), 6.91 (t, *J* = 7.3 Hz, 2H), 6.18 (dt, *J* = 3.6, 2.4 Hz, 1H) ppm. ^13^C NMR (176 MHz, DMSO-*d*_6_) δ 157.42, 157.15, 146.74, 131.35, 129.61, 124.24, 123.48, 119.68, 119.07, 116.60, 111.68, 109.50. HRMS calcd. C_12_H_11_N_3_O_2_ 229.0851, calcd. C_12_H_12_N_3_O_2_^+^ 230.0924, found M+H^+^ 230.0914.

#### (*E*)-N′-(2-hydroxy-4-methoxybenzylidene)-1H-pyrrole-2-carbohydrazide (2).

The precipitate was collected in 92% yield as white powder. ^1^H NMR (400 MHz, DMSO-*d*_6_) δ 11.72 (s, 1H), 11.63 (s, 1H), 11.60 (s, 1H), 8.45 (s, 1H), 7.41 (d, *J* = 6.9 Hz, 1H), 6.99 (s, 1H), 6.96 (s, 1H), 6.52 (d, *J* = 8.5 Hz, 1H), 6.49 (s, 1H), 6.18 (s, 1H), 3.77 (s, 3H) ppm. ^13^C NMR (176 MHz, DMSO-*d*_6_) δ 162.22, 159.62, 157.11, 147.34, 131.33, 124.68, 123.35, 112.48, 111.55, 109.52, 106.78, 101.64, 55.74. HRMS calcd. C_13_H_14_N_3_O_3_ 260.1035, found M+H^+^ 260.1023; calcd. C_13_H_13_N_3_O_3_Na 282.0844, found M+Na^+^ 282.0844.

#### (*E*)-N′-(4-(diethylamino)-2-hydroxybenzylidene)-1H-pyrrole-2-carbohydrazide (3)

The precipitate was collected in 95% yield as light yellow powder. ^1^H NMR (400 MHz, DMSO-*d*_6_) δ 11.66 (s, 1H), 11.45 (s, 1H), 11.40 (s, 1H), 8.34 (s, 1H), 7.18 (d, *J* = 7.5 Hz, 1H), 6.96 (s, 1H), 6.92 (s, 1H), 6.26 (d, *J* = 8.6 Hz, 1H), 6.16 (s, 1H), 6.12 (s, 1H), 1.10 (t, *J* = 6.6 Hz, 6H) ppm. ^13^C NMR (101 MHz, DMSO-*d*_6_) δ 159.92, 156.90, 150.36, 148.66, 131.76, 124.93, 123.00, 111.16, 109.42, 107.19, 104.05, 98.04, 44.26, 13.00. HRMS calcd. C_16_H_21_N_4_O_2_ 301.1659, found M+H^+^ 301.1657; calcd. C_16_H_20_N_4_O_2_Na 323.1478, found M+Na^+^ 323.1483.

#### (*E*)-N′-((2-hydroxynaphthalen-1-yl)methylene)-1H-pyrrole-2-carbohydrazide(4)

The precipitate was collected in 90% yield as yellow powder. ^1^H NMR (400 MHz, DMSO-*d*_6_) δ 12.85 (s, 1H), 11.87 (s, 1H), 11.85 (s, 1H), 9.40 (s, 1H), 8.22 (d, *J* = 8.6 Hz, 1H), 7.91 (t, *J* = 8.1 Hz, 2H), 7.61 (t, *J* = 7.7 Hz, 1H), 7.42 (t, *J* = 7.5 Hz, 1H), 7.24 (d, *J* = 8.9 Hz, 1H), 7.08–7.04 (m, 1H), 7.02 (s, 1H), 6.25 (d, *J* = 3.0 Hz, 1H). ^13^C NMR (176 MHz, DMSO-*d*_6_) δ 158.07, 157.00, 145.21, 132.73, 131.97, 129.42, 128.27, 128.11, 124.51, 123.94, 123.71, 121.02, 119.36, 111.69, 109.68, 109.17. HRMS calcd. C_16_H_13_N_3_O_2_ 279.1008, calcd. C_16_H_14_N_3_O_2_^+^ 280.1081, found M+H^+^ 280.1080; calcd. C_16_H_13_N_3_NaO_2_^+^ 302.0900, found M+Na^+^ 302.0909.

### UV-Vis Titration Experiments

A solution of sensors **1**-**4** (3 mM) in DMSO was prepared, and 10 μL sensors **1**-**4** (3 mM) was diluted with a 2.990 mL bis-tris buffer solution (PH = 7.4) to bring the final concentration to 10 μM. Then, 1-10 uL of Al(NO_3_)_3_ solution (3 mM) was added to the 3 mL of **1**-**4** solution (10 μM). After mixing for 1 min, UV-Vis absorption spectra were performed at room temperature.

### Fluorescence Titration Experiments

A solution of sensors **1**-**4** (3 mM) in DMSO was prepared, and 1 μL of sensors **1**-**4** (3 mM) was diluted with a 2.999 mL bis-tris buffer solution (PH = 7.4) to bring the final concentration to 1 μM. Then, 0.1-1 μL of Al(NO_3_)_3_ solution (3 mM) was added to the 3 mL of **1**-**4** solution (1 μM). After mixing for 1 min, fluorescence spectra were performed at room temperature.

### Job's Plot Measures

Bis-tris solutions of sensors 1-4 (10 μM) and Al(NO_3_)_3_ (10 μM) of the same concentration were prepared, respectively, and then a series of mixed solutions containing sensors 1-4 and Al(NO_3_)_3_ were prepared to keep the total volume of the solution at 3 mL. The volume ratio of the sensors and Al^3+^ ion are 10:0, 9:1, 8:2, 7:3, 6:4, 5:5, 4:6, 3:7, 2:8, 1:9, 0:10. After mixing for a few minutes, the fluorescence spectrum was acquired at room temperature. Job's Plot was drawn by plotting the fluorescence intensity and the ratio of sensor to ion volume.

### Competition Experiments

A solution of sensors **1**-**4** (3 mM) in DMSO was prepared, and 1 μL of sensors **1**-**4** (3 mM) was diluted with a 2.999 mL bis-tris buffer solution (PH = 7.4) to bring the final concentration to 1 μM. Solutions of various metal ions (4.5 μL, 20 mM, 30 equivalents) including Na^+^, K^+^, Ag^+^, Mg^2+^, Ca^2+^, Hg^2+^, Pb^2+^, Cd^2+^, Mn^2+^, Ni^2+^, Co^2+^, Cu^2+^, Zn^2+^, Fe^2+^, Fe^3+^, and Cr^3+^ were added to each sensor **1**-**4** solution (3 mL, 1 μM). The Al(NO_3_)_3_ 9H_2_O solution (4.5 mL, 20 mM, 30 equivalent) was then added to the above solutions. After mixing for 5 min, fluorescence spectra were taken at room temperature.

### ^1^H NMR Titration

Three NMR samples of sensor **3** (3.01 mg, 0.01 mmol) were dissolved in DMSO-*d*_6_ (500 μL), and three different amounts of Al(NO_3_)_3_ solutions (0, 0.5, and 1.0 eq.) were added to each sample. After the mixtures were mixed for 30 min, ^1^H NMR spectroscopy was performed at room temperature.

### Theoretical Calculation

DFT and TD-DFT calculations were operated by the Gaussian 09 software (Frisch et al., 2009) at the B3LYP/6–31G(d) level. The input files and orbital representations were generated with Gaussview 5.0 (scaling radii of 75%, isovalue of 0.05). Geometries and electronic properties were calculated by means of hybrid density functional B3LYP with the basis set of 6-31G(d). GaussSum 3.0 (O'Boyle et al., 2008) was used to calculate the contribution of molecular orbitals (MO) in electronic transitions and the theoretical absorption spectrum.

### Fluorescence Test Paper Detector

In order to facilitate rapid detection and analysis, a paper-based fluorescence sensor for Al^3+^ detection was developed. A series of circular test papers with a diameter of 0.5 cm were prepared by adding 10 μL of 10 μM sensor **3** solution, and then dried to make Al^3+^ test papers. Then 10 μL and 10 μM of different metal ions (Na^+^, K^+^, Ag^+^, Mg^2+^, Ca^2+^, Hg^2+^, Pb^2+^, Cd^2+^, Mn^2+^, Ni^2+^, Co^2+^, Cu^2+^, Zn^2+^, Fe^2+^, Fe^3+^, and Cr^3+^) were added to each test paper.

## Results and Discussion

### Synthesis of Sensors 1-4

**Scheme 2:**
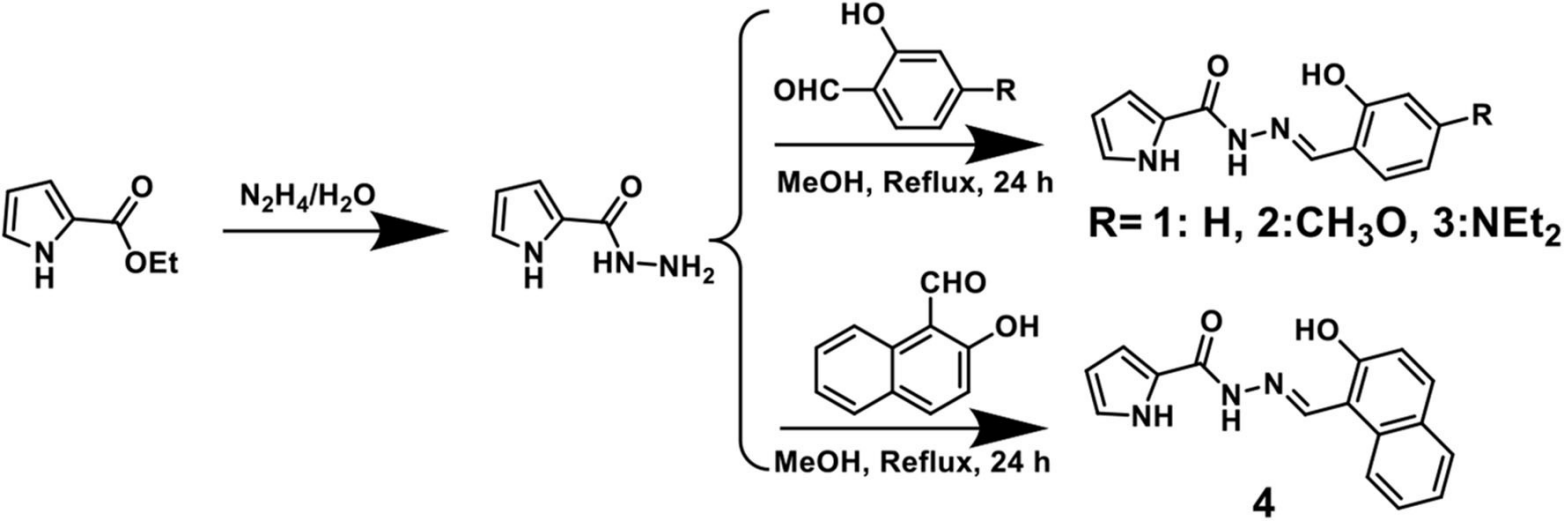
Synthetic route of sensors **1**-**4**.

**Figure 1:**
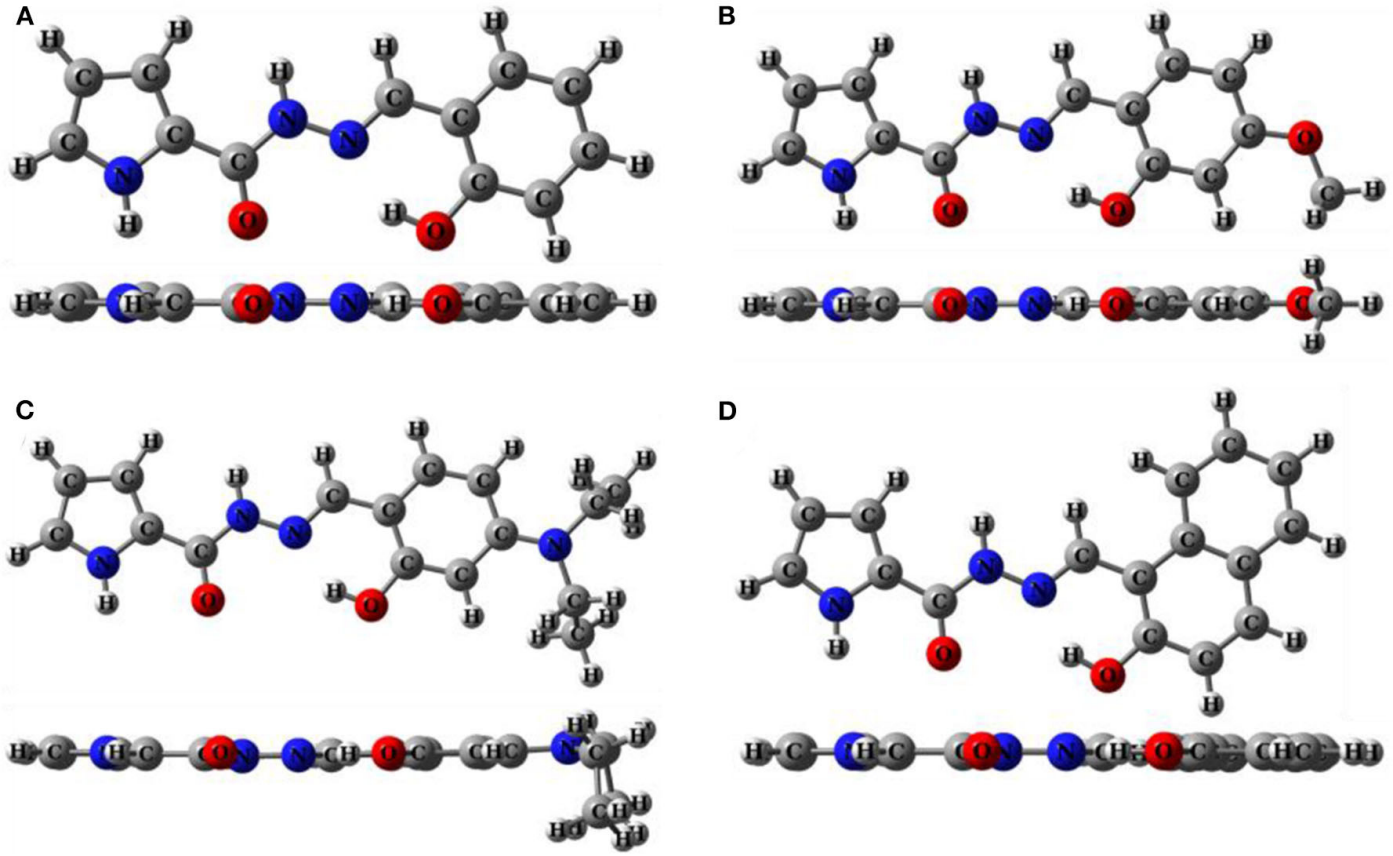
The up and side view of optimized molecular structure of **(A) 1**, **(B) 2**, **(C) 3**, **(D) 4**.

As shown in Scheme 2, sensors **1**-**4** were synthesized easily from o-hydroxybenzaldehyde derivatives and 1*H*-pyrrole-2-carbohydrazide via a one-step condensation reaction with high yields (≥ 90%). Sensors **1**-**4** have good solubility in many common solvents, such as methanol, ethanol, DMSO, and DMF, and it is also slightly soluble in water. These compounds were fully characterized by ^1^H NMR, ^13^C NMR, and HRMS (**Supplementary Figures 2–13**). The geometries of **1**-**4** were optimized by DFT calculations. The up and side views of the optimized molecular structure of **1**-**4** with atomic tags are shown in Figure 1. Due to the good conjugation in these molecules, the pyrrole ring, acylhydrazone, and benzene ring are almost on the same plane, which makes them have excellent planarity, and it is conducive to the intramolecular PET effect. Therefore, **1**-**4** aqueous solutions showed very weak fluorescence intensity.

### UV-Vis Absorption Studies

**Figure 2:**
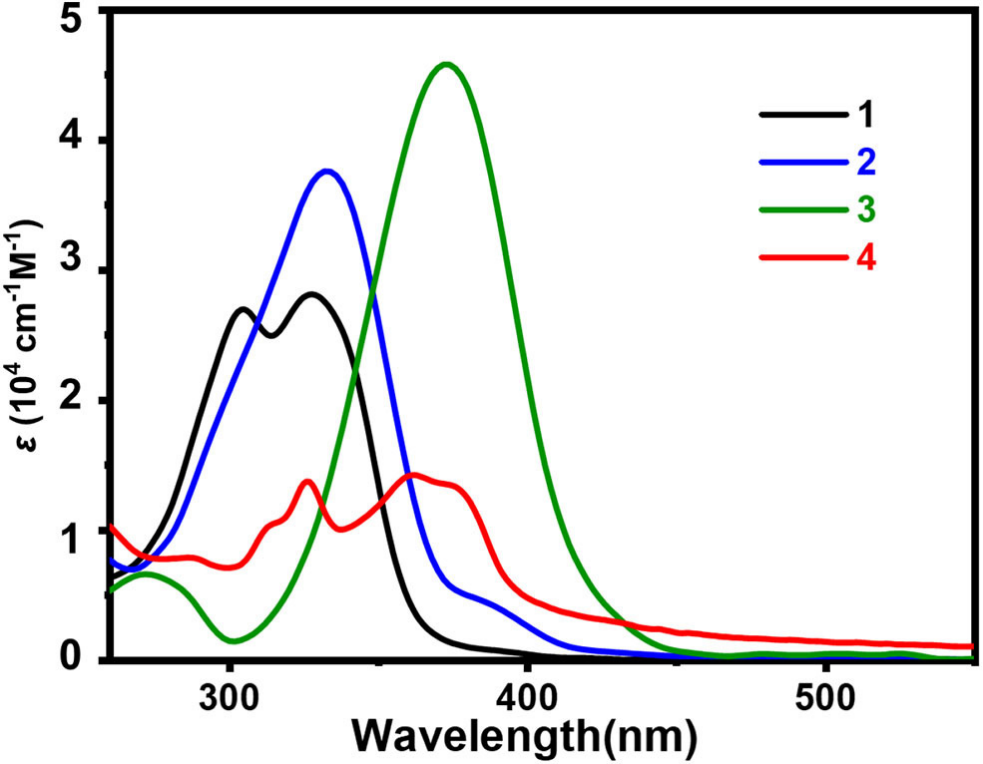
UV-vis absorption spectrum of 1-4 (10 μM) in 0.3% DMSO/bis-tris buffer solution (PH = 7.4).

In the research for the investigation of sensing property, the absorption spectrum of sensors is an essential photophysical property. All of the absorption spectra were recorded in aqueous solution (about 0.3% DMSO) at room temperature. The data are summarized and shown in Figure 2 and Table 1. The UV-Vis absorption spectrum of compounds **1**-**4** in aqueous solution (0.3% DMSO) is shown in Figure 2. The figure shows two main absorption bands, corresponding to π–π^*^ and n–π^*^ transitions, respectively. The maximum absorption peaks of compounds **1**-**4** are 328, 333, 372, and 376 nm, respectively. Compared with **1**, the maximum absorption peaks of compounds **2-4** all show clear red shifts. However, the maximum absorption peaks of compounds **3** and **4** show large red shifts (44 and 48 nm, respectively), this may due to the additional donor group (N, N-diethyl) (**3**) and extended π conjugation (**4**). The absorption spectrum of **2** only shows a small amount of red shift (5 nm) owing to the electron donating ability of the methoxy group is weaker than that of the N, N-diethyl group. As shown in the absorption spectrum obtained by theoretical calculations of **1**-**4** (**Supplementary Figure 22**), the maximum absorption peaks of compounds **1**-**4** are 334, 339, 370, and 373 nm. Compared with **1**, the maximum absorption peaks of **2**, **3**, and **4** show obvious red shifts. The amounts of red shift are almost the same as the data obtained by the experiment.

**Table 1:** Photophysical data of 1-4 with and without Al^3+^ in aqueous solution.

**Compound**	**Absorption^**a**^**	**Emission**^**b**^	
	**λ_**max**_ (nm), ε (10^**4**^ cm^**−1**^M^**−1**^)**	**λ_**max**_ (nm)**	**Φ**
1	328 (2.7)	429	0.012
2	333 (3.8)	430	0.015
3	372 (4.6)	445	0.011
4	376 (1.3)	472	0.042
1-Al	387 (2.1)	446	0.199
2-Al	395 (2.4)	448	0.314
3-Al	417 (2.6)	453	0.315
4-Al	425 (1.2)	480	0.093

a Measured in 0.3% DMSO/bis-tris buffer solution (PH = 7.4) at 1.0 × 10^−5^ M, RT.

b *Recorded in 0.03% DMSO/bis-tris buffer solution (PH = 7.4) at 1.0 × 10^−6^ M*.

**Figure 3:**
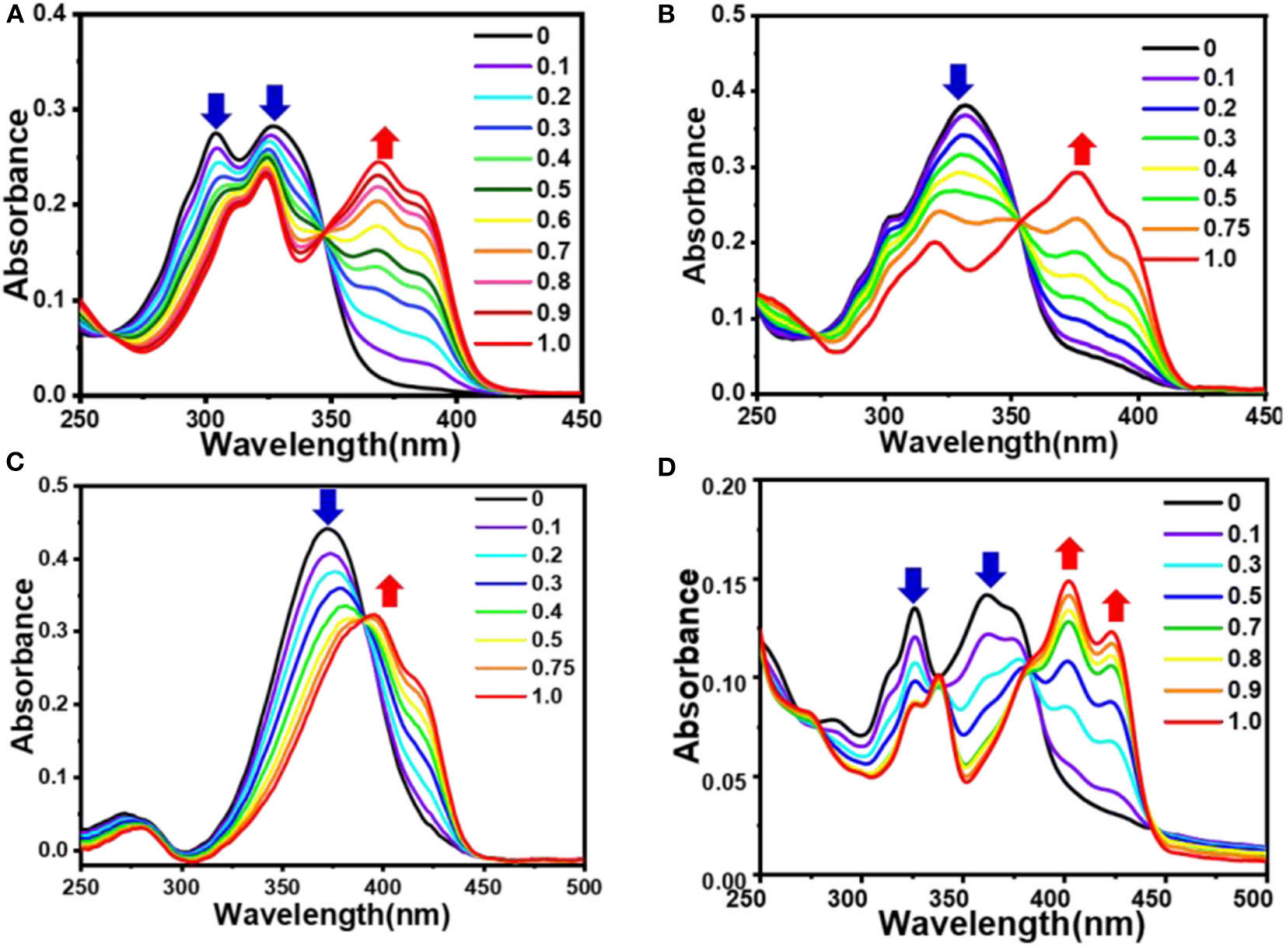
Absorption spectra of **1 (A), 2 (B), 3 (C), 4 (D)** (10 μM) upon the addition of Al^3+^(0-1 eq.) in 0.3% DMSO/bis-tris buffer solution (PH = 7.4).

UV-vis titration analysis was performed by gradually adding Al^3+^ ions (0-1 eq.) to compounds **1**-**4**, respectively. As shown in Figure 3, each compound absorption band has a significant change with the gradual increase of Al^3+^ ion content. Compound **1** has two new absorption bands near 325 nm and 375 nm, and absorption bands near 300 and 325 nm gradually decreasing, there are two equivalent absorption points at 256 and 350 nm; **2** has two new absorption bands near 310 and 380 nm, the absorption band near 333 nm gradually decreases, the equal absorption points are at 275 and 360 nm; **3** has two new absorption bands at 275 and 398 nm, the absorption band near 372 nm gradually decreases with an equivalent point near 390 nm; and **4** has three new absorption bands at 340, 402, and 425 nm. The absorption bands around 325 and 376 nm gradually decrease, and the equivalent absorption points are at 276 and 380 nm, respectively. These bands are attributed to the π-π^*^ transition of the pyrrole unit and the charge transfer transition in the Al^3+^ complexes. The existence of the equivalent absorption points indicates that coordination interactions exist between the ligands **1**-**4** and Al^3+^ ion.

### Interaction Between Sensors and Al^3+^

With the addition of Al^3+^ from 0 to 1.0 eq., the new absorption peaks of compounds **1**-**4** at 372 nm, 385, 398, and 425 nm increase linearly (Figure 3). This result indicates that **1**-**4** can be used for quantitative analysis of Al^3+^. When the amount of Al^3+^ added exceeds 1 eq., the weak increase in peak intensity indicates that the binding mode of the sensors **1**-**4** toward Al^3+^ follows a 1:1 stoichiometric ratio.

According to the calculation of equation LOD > 3σ/k, the detection limits of sensors **1**-**4** for Al^3+^ are 53, 45, 42, and 102 nM, where σ is the standard deviation of the blank measurement, and k is the slope of the calibration line (**Supplementary Figure 16**). Using the Benesi-Hildebrand expression, the association constants (K_a_) of **1**-**4** were calculated to be approximately 4 × 10^4^, 5.3 × 10^4^, 4.5 × 10^4^, and 1.1 × 10^4^ (**Supplementary Figure 17**). The comparative analysis of sensors **1**-**4** with previously reported sensors are shown in **Supplementary Table 1**.

**Figure 4:**
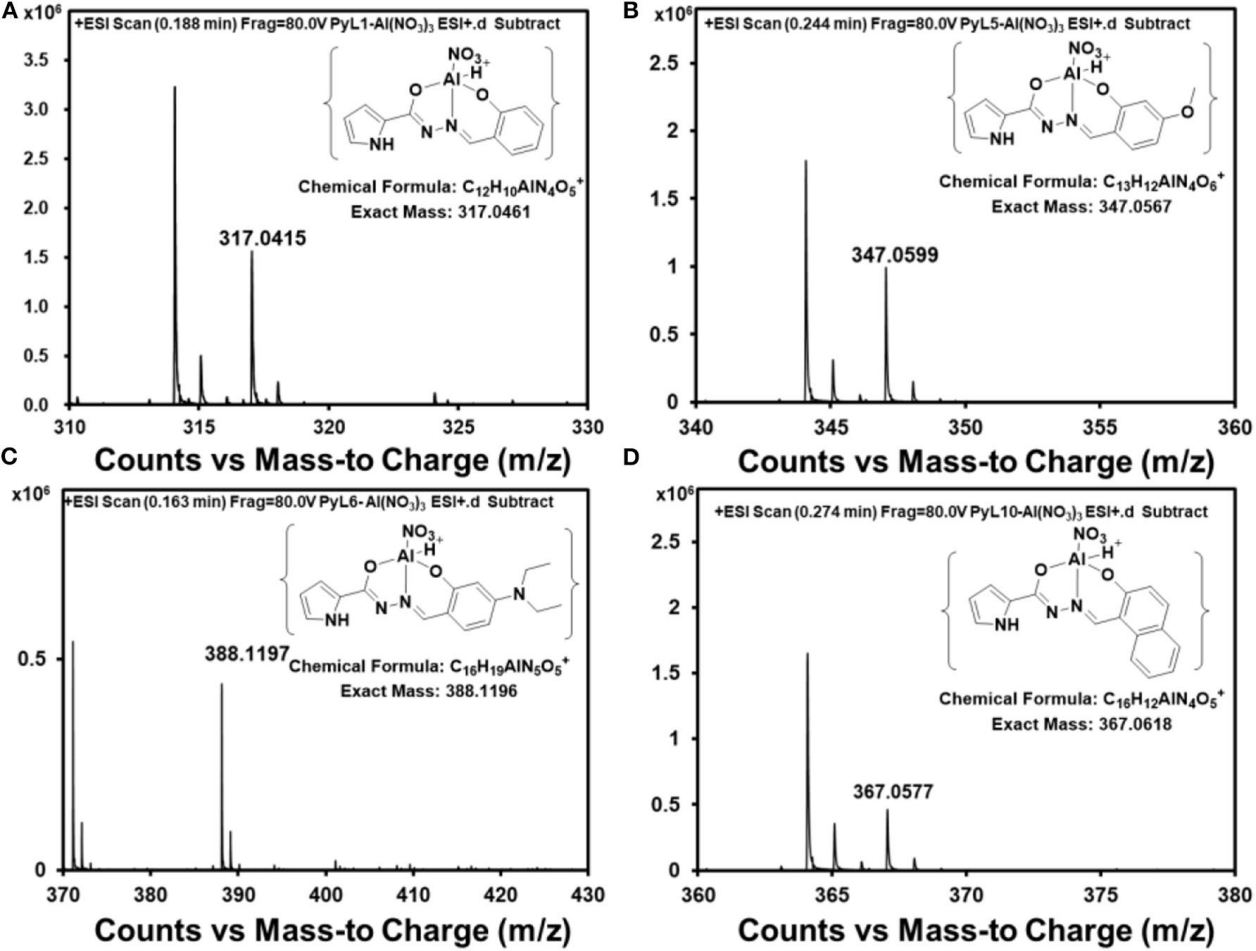
ESI-MS spectrum of **1**-Al **(A)**, **2**-Al **(B)**, **3**-Al **(C)**, **4**-Al **(D)**.

In order to confirm the binding mode of **1**-**4** toward Al^3+^, Job's Plot for fluorescence was carried out (**Supplementary Figure 18**). The maximum fluorescence intensity of **1**-**4** were reached at a molar fraction of 0.5, indicating a 1:1 ratio for the Al^3+^ complexes of **1**-**4**. Furthermore, the ESI mass spectra of **1**-**4** in the presence of Al^3+^ were also measured (Figure 4), which is regarded as direct evidence for understanding the binding mode between metal ions and sensors. The ESI-MS peaks at m/z 317.0415, 347.0599, 388.1197, and 367.0618, corresponding to [AlNO_3_·(**1**-H)]^+^ (calcd = 317.0461), [AlNO_3_·(**2**-H)]^+^ (calcd = 347.0567), [AlNO_3_·(**3**-H)]^+^ (calcd = 388.1196), and [AlNO_3_·(**4**-H)]^+^ (calcd = 367.0618) can be clearly observed when an excess amount of Al^3+^ were added to **1**-**4** aqueous solutions, suggesting a 1:1 Ligand-Al^3+^ binding stoichiometry (Figure 4). It can be seen from the results that each sensor was combined to an aluminum atom with a nitrate.

### ^1^H NMR Titrations

**Figure 5:**
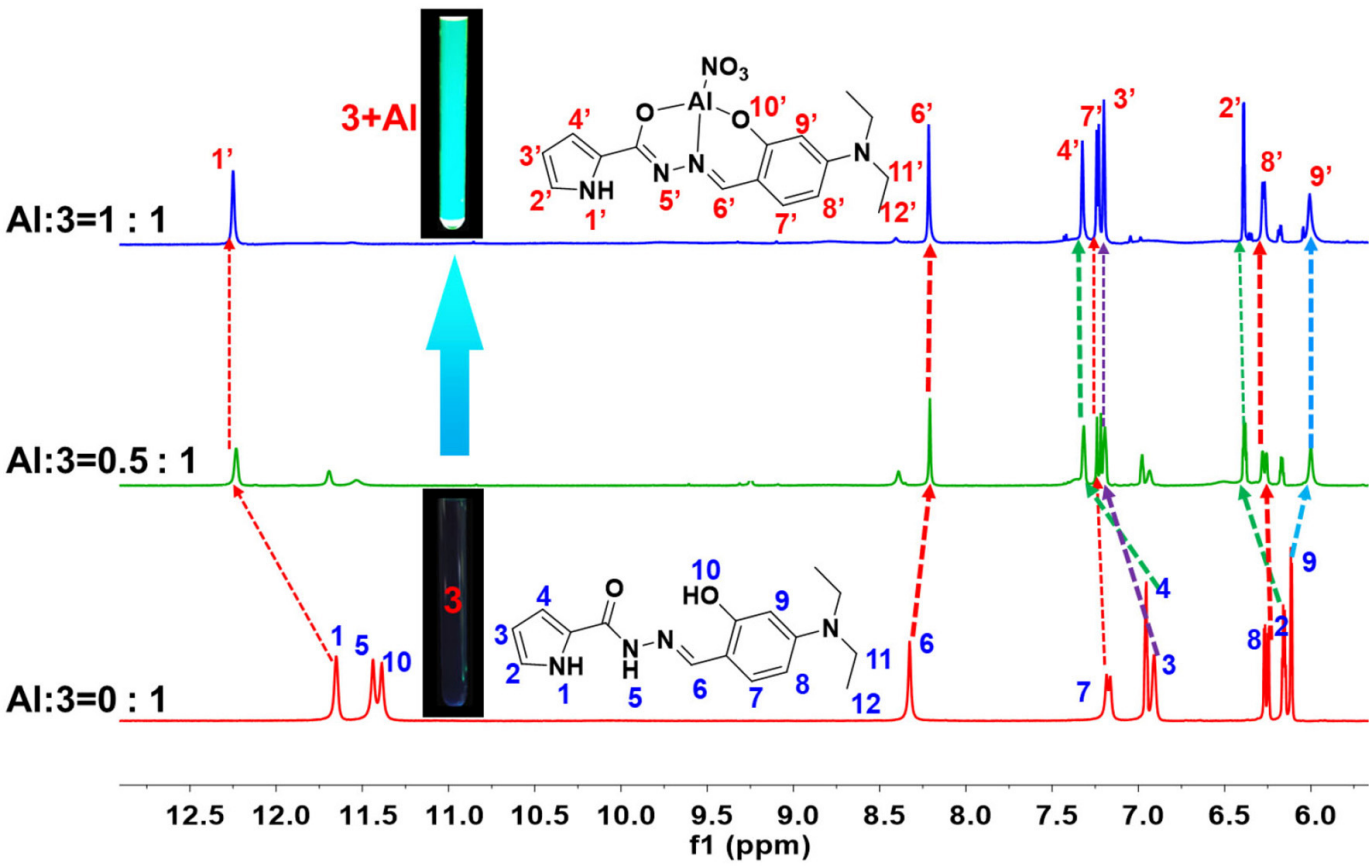
^1^H NMR titration of **3** upon adding 0-1 eq. of Al^3+^ in DMSO-*d*_6_ (Inset: Photograph of **3** and **3**-Al in NMR tube under the irradiation of a 365 nm UV lamp).

In order to further study the absorption and fluorescence phenomena of the sensors, ^1^H NMR titration was performed by adding Al^3+^ to sensor **3** (0.5 mM in DMSO–*d*_6_ solution). Firstly, ^1^H-^1^H COSY NMR spectrum of sensor **3** was performed assign the chemical shift of the hydrogen atoms (**Supplementary Figure 14**). The chemical shift of N-H(H1) on the pyrrole ring was 11.69 ppm. The chemical shift of N-H (H5) was 11.40 ppm, and the chemical shift of hydroxyl O-H (H10) on the benzene ring was 11.39 ppm. After adding Al^3+^ to the sensor **3** by 0.5 equivalent, it can be seen that the ^1^H NMR spectrum significantly changed (Figure 5). The chemical shift signals of N-H (H5) on the hydrazide group and O-H (H10) of the hydroxyl group on the benzene ring gradually weakened. After the addition of Al^3+^ increasing to 1 equivalent, the shift signals of H5 and H10 disappeared completely. This also verified that the binding stoichiometry of **3** to Al^3+^ was 1:1. By testing the ^1^H-^1^H COSY NMR spectrum of **3**-Al^3+^ (**Supplementary Figure 15**), the signal peaks of each hydrogen of the titration product can be assigned. Through the titration process, we can observe that the chemical shift of H1 migrates from 11.69 ppm to low field 12.23 ppm, H6 chemical shift from 8.37 ppm to high field 8.26 ppm, H2, H3, H4, H7, H8, H9 chemical shift from 6.20, 6.95, 7.00, 7.21, 6.30, and 6.15 ppm to 6.38, 7.20, 7.32, 7.23, 6.26, and 6.00 ppm. The disappearance of the strong N-H spike indicates that it participates in the enol interconversion of the carbonyl group, and the disappearance of the proton peak of phenol-OH indicates the interaction between Al^3+^ and phenol-OH. The overall change of the ^1^H NMR spectrum indicates that Al^3+^ and **3** are complexed and coordinated through the O atom of the carbonyl group, the azomethine-N atom, and the phenolic-OH oxygen atom.

### Fluorescence Studies

**Figure 6:**
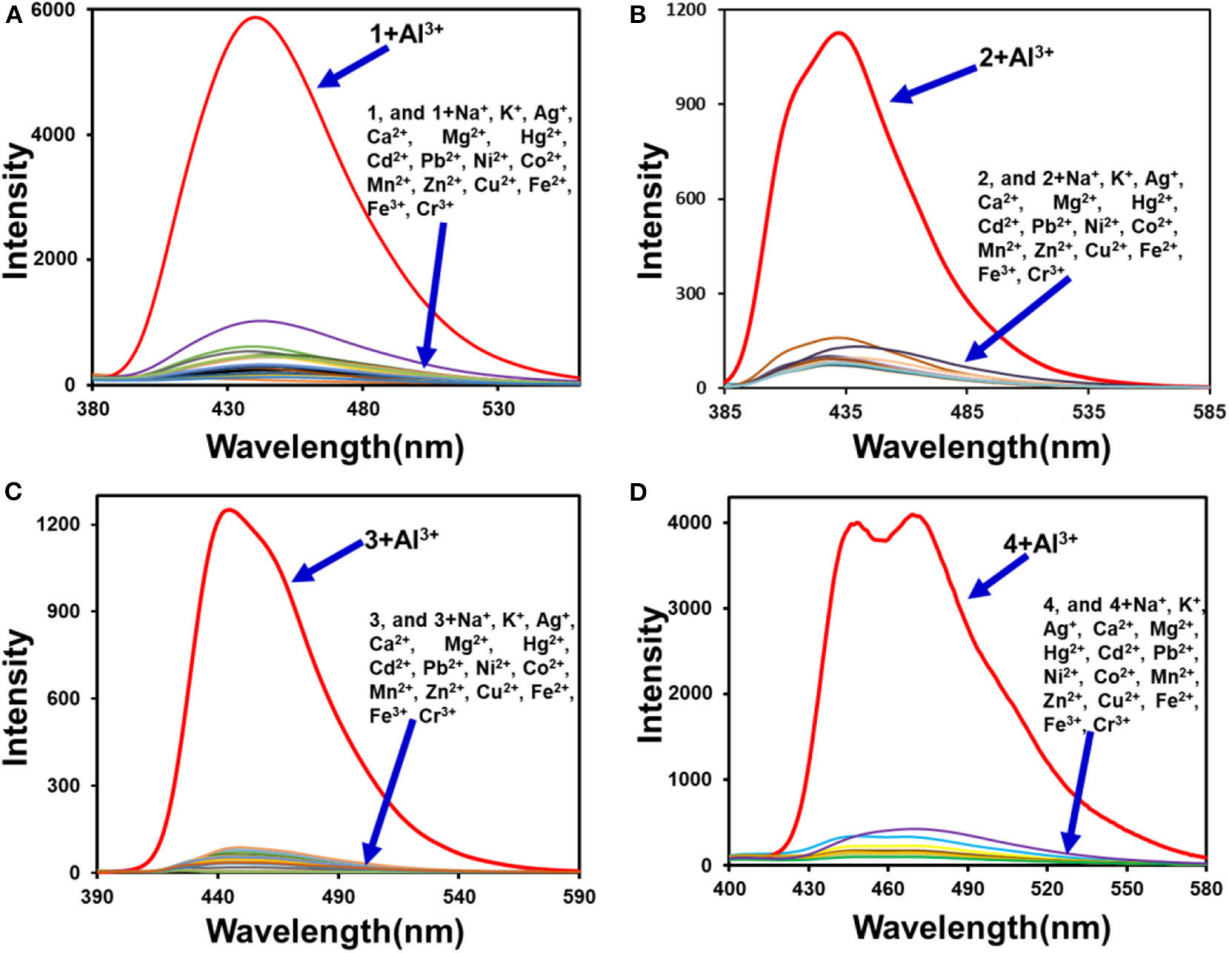
Fluorescence spectra of 1 **(A)**, 2 **(B)**, 3 **(C)**, 4 **(D)** (1 μM) with metal ions (30 μM) (Na^+^, K^+^, Ag^+^, Mg^2+^, Ca^2+^, Hg^2+^, Pb^2+^, Cd^2+^, Mn^2+^, Ni^2+^, Co^2+^, Cu^2+^, Zn^2+^, Fe^2+^, Fe^3+^, Cr^3+^, and Al^3+^).

**Figure 7:**
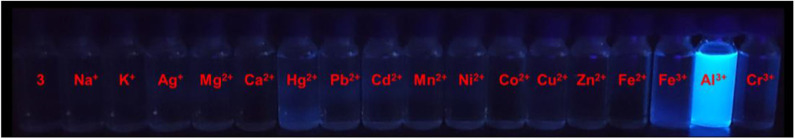
Fluorescence photo of **3** (1 μM) and **3** with metal ions (1 μM) (Na^+^, K^+^, Ag^+^, Mg^2+^, Ca^2+^, Hg^2+^, Pb^2+^, Cd^2+^, Mn^2+^, Ni^2+^, Co^2+^, Cu^2+^, Zn^2+^, Fe^2+^, Fe^3+^, Cr^3+^, and Al^3+^) under the irradiation of an 365 nm UV lamp.

**Figure 8:**
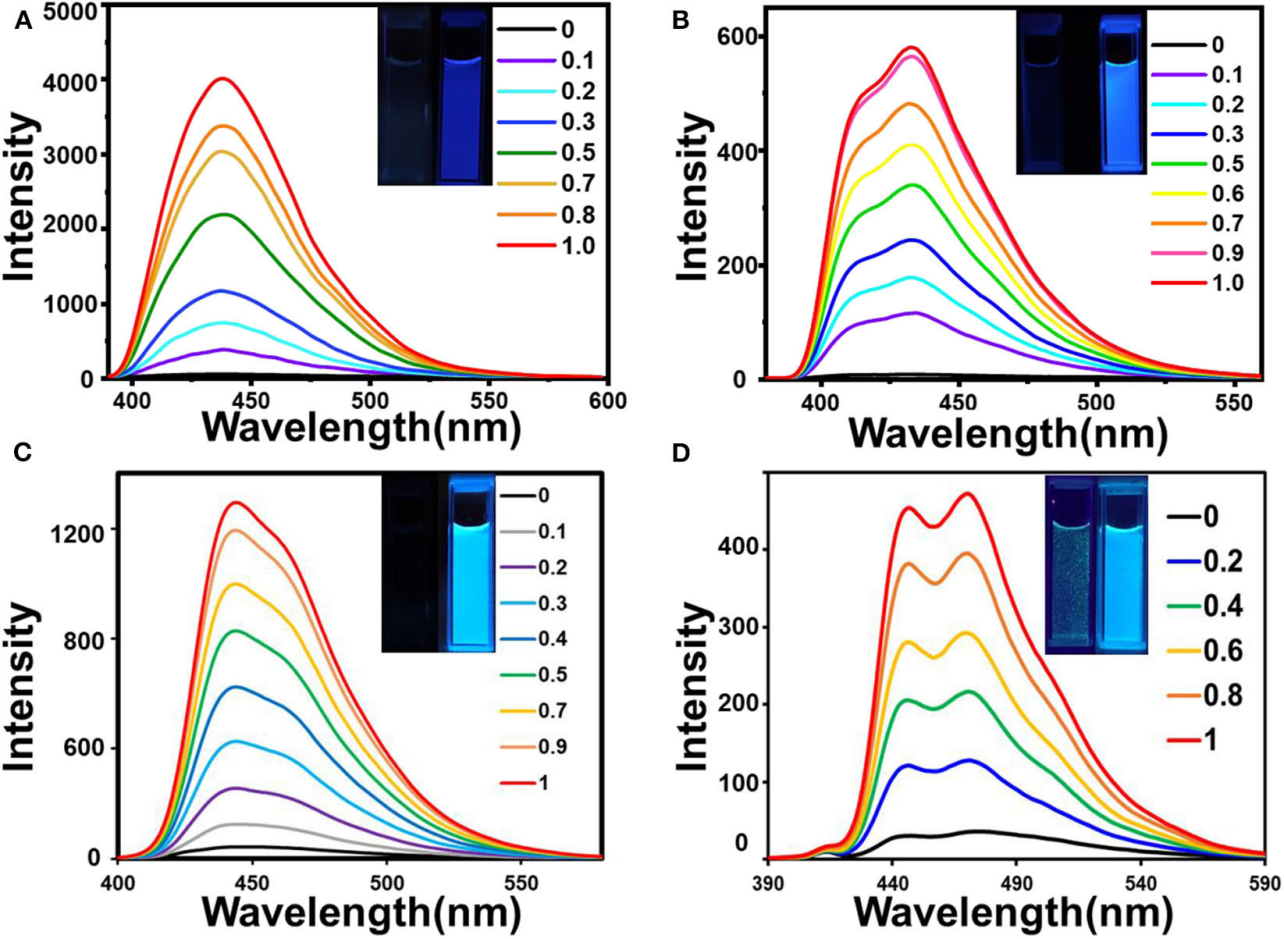
Fluorescence spectra of **1 (A), 2 (B), 3 (C), 4 (D)** (1 μM) upon addition of different equivalent Al^3+^ in 0.03% DMSO/tris-bis buff solution (insets: comparison photos of **1**-**4** and their Al^3+^ complexes under a 365 nm UV lamp irradiation).

Fluorescence emission studies were conducted to check the selectivity of **1**-**4** for various metal ions (Na^+^, K^+^, Ag^+^, Mg^2+^, Ca^2+^, Hg^2+^, Pb^2+^, Cd^2+^, Mn^2+^, Ni^2+^, Co^2+^, Cu^2+^, Zn^2+^, Fe^2+^, Fe^3+^, Cr^3+^, and Al^3+^) (Figure 6, **Supplementary Figure 19**). After adding the Al^3+^ aqueous solution, the fluorescence intensity of the system was significantly enhanced. However, metal complexes of 1-4 still showed weak fluorescent emission with the addition of other kinds of metal ions. In order to see the fluorescence changes more intuitively, we prepared multiple **1**-**4** samples with different metal ions. After the addition of Al^3+^ ions, Al^3+^ complexes of **1**-**4** emitted strong fluorescence under the irradiation of an UV lamp. Compared with **1**-**4**, the fluorescence intensity of their Al^3+^ complexes had changed significantly, however, there is no obvious change of fluorescence intensity when other metal ions were added to **1**-**4** (Figures 7, 8, **Supplementary Figure 20**). Therefore, sensors **1**-**4** are highly selective for the detection of Al^3+^.

The fluorescence titrations of sensors **1**-**4** were conducted with increasing amounts of Al^3+^ (Figure 8). It shows that with the addition of Al^3+^ ions (0-1 eq), the fluorescence intensity of **1**-**4** increased linearly at 446, 448, 453, and 480 nm, respectively. Due to the PET effect and ESIPT processes, ligands **1**-**4** emit very weak fluorescence. The fluorescence quantum yields of **1**-**4** (1 μM) with and without Al^3+^ are shown in Table 1. The quantum yields of **1**-**4** are 0.012, 0.015, 0.011, and 0.042. The quantum yields of **1-Al**-**4-Al** are 0.199, 0.314, 0.315, and 0.093, respectively. The quantum yields changed significantly before and after the sensors **1**-**4** binding to Al^3+^. Especially sensors **2** and **3**, where the fluorescence quantum yield was enhanced by more than 20 times.

In order to verify the preferential selectivity of **1**-**4** for Al^3+^, various competition experiments were conducted to interfere with metal ions. As shown in **Supplementary Figure 21**, most of the metal ions have little influence on the detection of Al^3+^ by the detectors **1**-**4**, and only a small part of the metal ions have influence on the Al^3+^ detection process. Hg^2+^, Cu^2+^, Fe^2+^, and Fe^3+^ have weak effects on the detection process accompanied by a decrease in fluorescence intensity. The interference to Hg^2+^, Cu^2+^, Fe^2+^, and Fe^3+^ may be caused by their intrinsic quenching properties to fluorescence (Jeong et al., 2017).

### Theoretical Calculation

**Figure 9:**
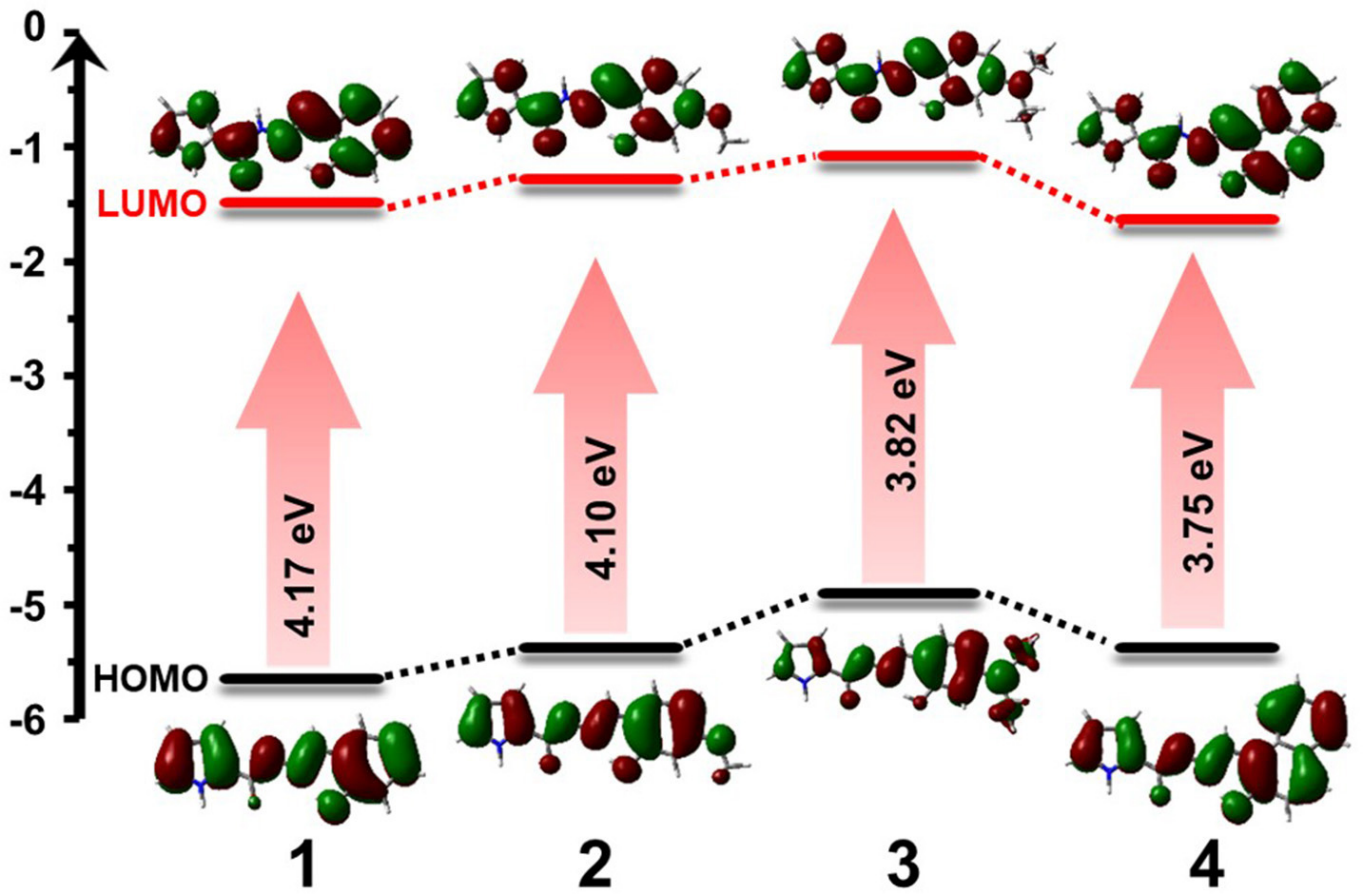
HOMO and LUMO orbital levels and HOMO-LUMO energy gap of **1**-**4**, calculated with DFT at the B31LYP/6-31G level using Gaussian 09.

In order to more clearly understand the spectral change behavior among **1**-**4**, TD-DFT calculation was operated based on B3LYP/6-31G(d). From TD-DFT data, HOMO and LUMO energy levels and the corresponding electron cloud distribution map of each compound were obtained. HOMO energy levels of **1**-**4** were −5.65, −5.38, −4.90, and −5.38 eV, and the LUMO energy levels were −1.48, −1.28, −1.08, and −1.63 eV (Figure 9, **Supplementary Tables 2–5**). Therefore, through the formula *E*_gap_ = *E*_LUMO_ – *E*_HOMO_, it is easy to obtain the HOMO-LUMO energy gap (*E*_gap_) values of **1**-**4** as 4.17, 4.10, 3.82, and 3.75 eV, respectively. Comparing **2**, **3**, and **1**, it can be seen that after introducing the electron-donating substituents N, N-diethyl and methoxy groups on the benzene ring, the energy levels of HOMO and LUMO had improved, but the increase of HOMO was greater than that of LUMO. Therefore, the *E*_gap_ of **2**, **3** was narrowed. Comparing **4** and **1**, it can be seen that after the benzene ring was replaced with a naphthalene ring, the π-conjugation of the molecule expanded. The LUMO energy level was greatly reduced and the HOMO energy level increased, thus narrowing the *E*_gap_. The narrower the *E*_gap_ of the molecule was, the more favorable it would be for the internal electrons of the molecules to be excited. Thus, a red shift in the absorption spectrum occurred. The *E*_gap_ gradually becomes smaller from **1** to **4**, and the maximum absorption wavelength in the absorption spectrum obtained by the calculation gradually becomes larger, which coincides with the experiment results (**Supplementary Figure 22**, **Supplementary Table 6**).

**Figure 10:**
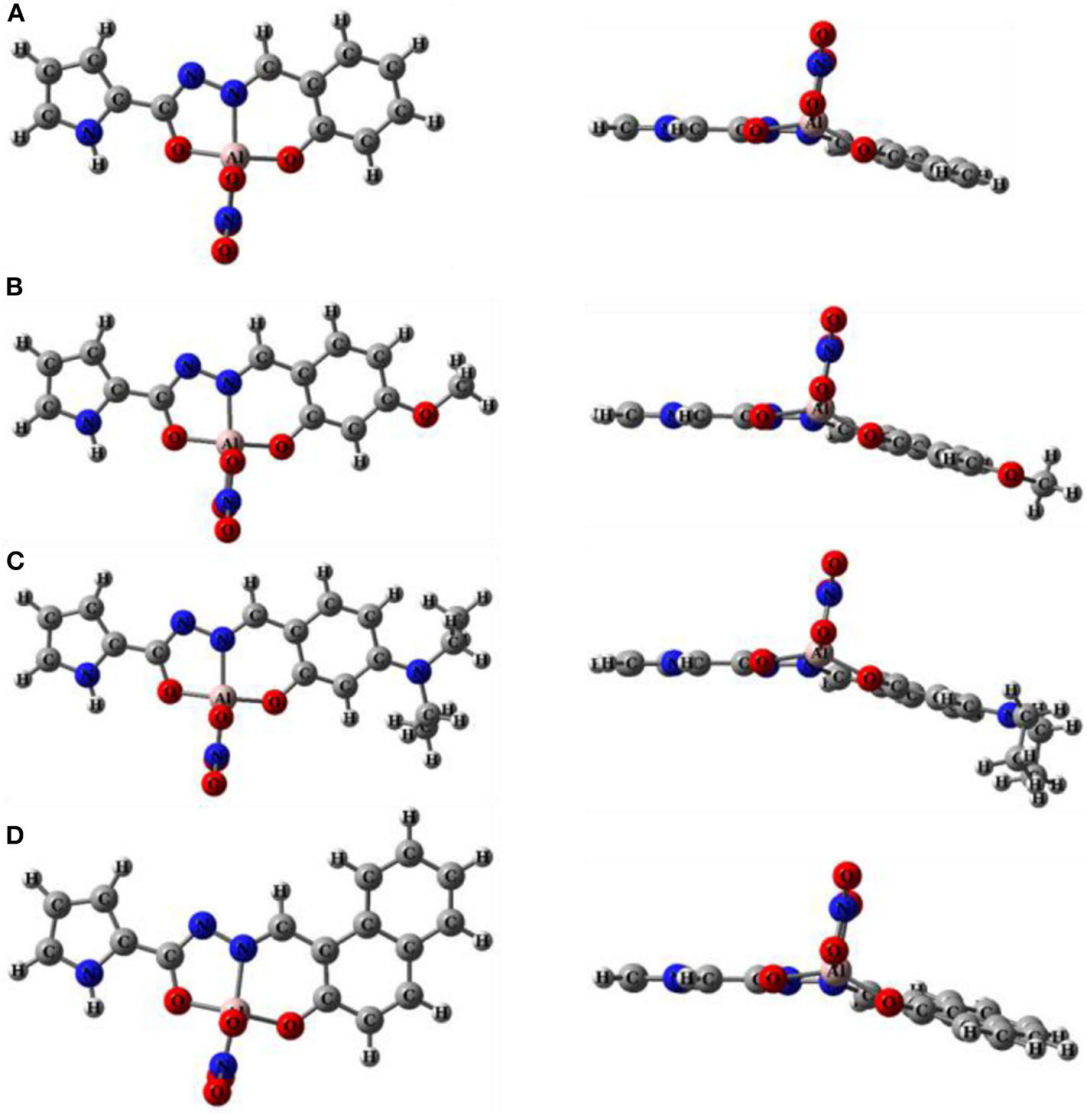
Up view and side view of optimized molecular structure of **(A) 1**-Al, **(B) 2**-Al, **(C) 3**-Al, **(D) 4**-Al.

The possible binding mode of sensors and Al^3+^ has been verified by Job's plot, ESI-MS, and ^1^H NMR titration, that is, the sensors and Al^3+^ are combined through a 1:1 coordination ratio. DFT and TD-DFT calculations were conducted to optimize the possible molecular structures of **1**-Al, **2**-Al, **3**-Al, and **4**-Al based on B3LYP/6-31G (d) (Figure 10). From the calculation results, the aluminum atom was coordinated with one N and two O in the ligand, and a nitrate was also connected to the aluminum atom.

**Figure 11:**
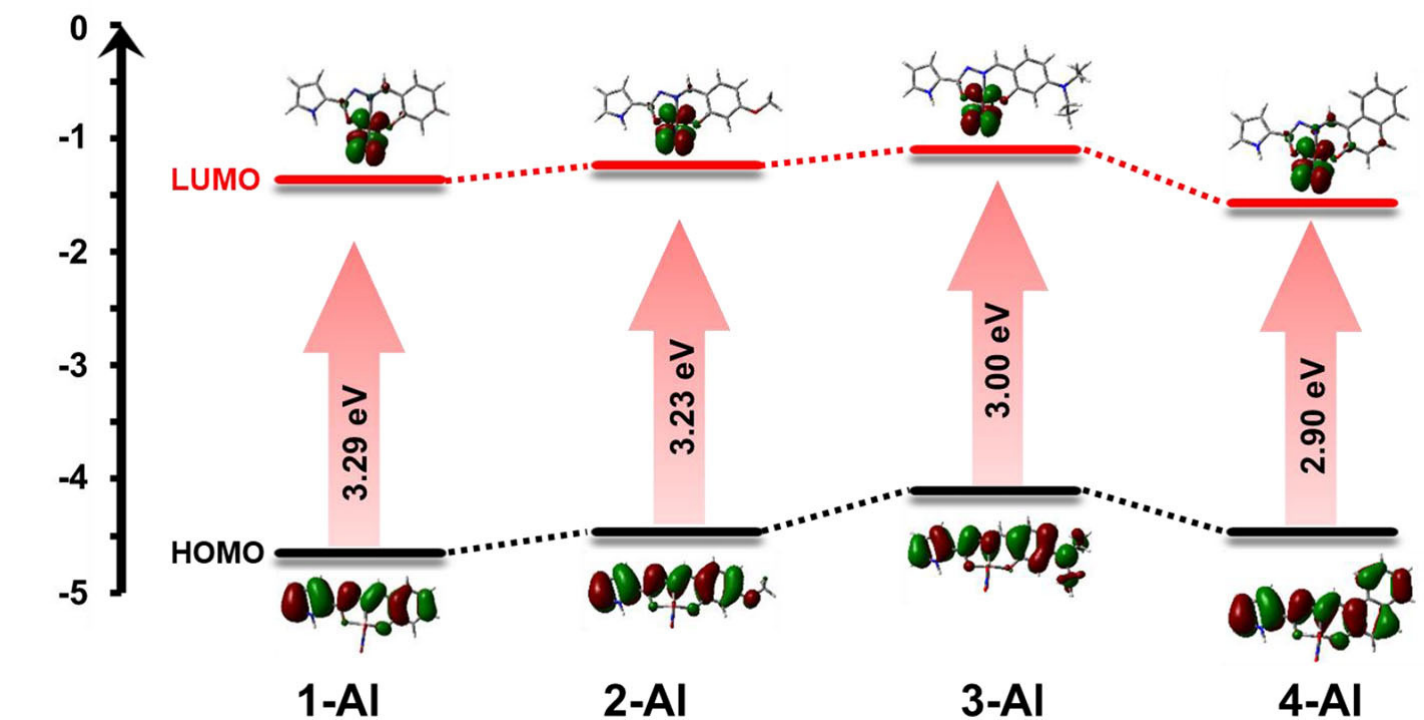
HOMO and LUMO orbital levels and HOMO-LUMO energy gap of **1**-Al, **2**-Al, **3**-Al, and **4**-Al, calculated with DFT at the B31LYP/6-31G level using Gaussian 09.

From the structure of the sensors **1**-**4**, they are mainly divided into two parts, pyrrolyl hydrazide and salicylaldehyde derivatives. It can be seen that the HOMO orbital electron cloud of **1**-Al is mainly distributed on the ligand π conjugated skeleton, and the LUMO orbital electron distribution is mainly concentrated on the Al atom (Figure 11). Sensors **2**-**4** have the same orbital distribution as **1**. The *E*_gap_ of sensor **1**-Al is 3.29 eV. The *E*_gap_ for sensor **2**-Al, **3**-Al, and **4**-Al are 3.23, 3.00, and 2.90 eV, respectively. The *E*_gap_ has decreased significantly from sensor **1**-**4** to **1**-Al - **4**-Al, indicating the *E*_gap_ becomes narrower during the combination process of probe and Al^3+^, which means that it takes less energy to excite Al complexes than the ligands. As for the experimentally obtained absorption spectrum, it can be seen that the maximum absorption peak red shift occurs after the sensors combined with Al^3+^, it needs lower energy to excite the system to emit fluorescence, which is consistent with the conclusions obtained from the theoretical calculations. The electron cloud of HOMO in sensors-Al^3+^ are mainly distributed on the π conjugate skeleton of the ligand, and the LUMO is mainly concentrated on the central metal Al, the possible reason is mainly the electronic transition caused by LMCT.

### “Turn-On” Mechanism of the Sensors

**Figure 12:**
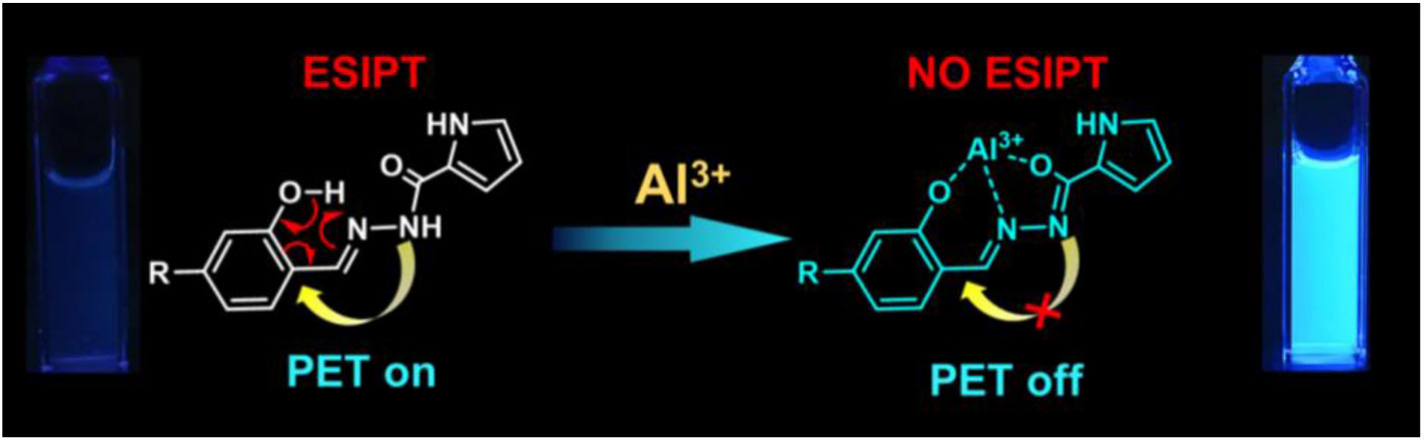
Proposed sensing mechanism of sensors to Al^3+^.

From the side view of the optimized molecular structure diagram (Figures 1, 10), it can be clearly seen that after the sensors bind to the Al^3+^ ion, the coplanarity of the pyrrole ring and the salicylaldehyde ring of the sensors is destroyed, which is not conducive to the charge transfer. The combination of Al atoms and carbonyl oxygen atoms also destroy the enol interconversion of the carbonyl group at the hydrazide structure in the molecule which inhibits the PET and ESIPT effect (Figure 12).

### Application of Al^3+^ Test Paper

**Figure 13:**
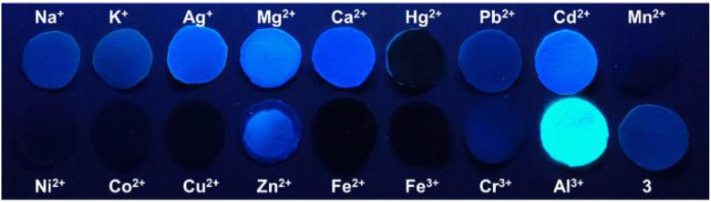
Photograph of detecting metal ions with the test paper of sensor **3**, and test by dropping different ions onto the test paper of sensor **3**. The image was obtained under the irradiation of a 365 nm UV lamp.

In order to explore the practical application of the sensors, and make them easily and conveniently applied in life, we have developed test paper for Al^3+^ detection based on **3**. A series of 3.0 μL 0.5 mM sensor **3** solutions were dropped onto the round filter papers and then dried to obtain blue-violet fluorescent test papers. To each test paper, 1 μL of various metal ions solutions were added dropwise. After a few seconds, under the ultraviolet light, we could see that the test paper with Al^3+^ emitted a strong blue-green fluorescence (Figure 13). The fluorescence of test papers with the addition of other metal ions were almost unchanged. We also used a paper-made pen which was dipped in Al^3+^ solution to write on a test paper. We could clearly see that the element symbol “Al” emitted strong blue-green fluorescence under the irradiation of a 365 nm UV lamp (**Supplementary Figure 24**). The simple and easy-to-prepare test paper made by sensor **3** had high selectivity and high sensitivity for identifying Al^3+^.

## Conclusions

In conclusion, we have synthesized and characterized four novel biosensors (**1**-**4)** based on pyrrole hydrazone Schiff bases. These sensors have outstanding “turn-on” fluorescence recognition for Al^3+^ ions in aqueous solutions. The binding modes of **1**-**4** with Al^3+^ were determined by Job's Plot and ESI-MS spectrometry. The detection limits of **1**-**4** for Al^3+^ were 53, 45, 42, and 102 nM. ^1^H-^1^H COSY NMR spectrum and ^1^H NMR titration have also been studied for the interaction between **3** and Al^3+^. Moreover, DFT and TD-DFT theoretical calculations were used for molecular structure optimization, HOMO, LUMO, and *E*_gap_ calculations and comparisons. It was also used to explain the spectral difference among ligands **1**-**4**, as well as the change of absorption spectrum before and after the interaction between **1**-**4** and Al^3+^ and the fluorescence mechanism. The sensing mechanisms were proposed with the PET and ESIPT effect. A high selectivity test strip was developed for Al^3+^ detection-based sensor **3**. On the basis of the results, we believe that the results will provide an important reference for the development and application in biosensors fields.

## Data Availability Statement

The original contributions presented in the study are included in the article/Supplementary Materials, further inquiries can be directed to the corresponding author/s.

## Author Contributions

PW and HL designed the work. PW did the experiment and wrote the manuscript. LL, FM, MK, and HL revised and edited the manuscript. All authors reviewed the manuscript and have agreed to its publication.

## Conflict of Interest

The authors declare that the research was conducted in the absence of any commercial or financial relationships that could be construed as a potential conflict of interest.
