# Supplementary material for: “Turn-On” Fluorescent Biosensors for High Selective and Sensitive Detection of Al3+ Ion
Source: Front Chem. 2020 Nov 19;8:607614. doi: 10.3389/fchem.2020.607614 (PMC7711066; doi:10.3389/fchem.2020.607614)
Supplement: Supplementary file 1 [file Table_1.DOCX]

Supplementary Material

“Turn-on” Fluorescent Biosensors for High Selective and Sensitive Detection of Al^3+^ ion

Pengfei Wang ^1^, Lijie Liu ^1^, Fanda Meng ^2^, Maroof Ahmad Khan ^1^, Hui Li ^1*^

^1^Key Laboratory of Cluster Science of Ministry of Education, School of Chemistry and Chemical Engineering, Beijing Institute of Technology, Beijing 100081, P. R. China.

^2^ Institute of Basic Medicine, Shandong First Medical University & Shandong Academy of Medical Sciences, Shandong, P. R. China.

*** Correspondence:**Hui Li
E-mail: [lihui@bit.edu.cn](mailto:lihui@bit.edu.cn)

**Table of Contents**:

Figure **S1**. ^1^H NMR (400 MHz, DMSO-*d*_6_) spectrum of **1*H*-pyrrole-2-carbohydrazide**.

Figure **S2**. ^1^H NMR (400 MHz, DMSO-*d*_6_) spectrum of **1**.

Figure **S3**. ^1^H NMR (400 MHz, DMSO-*d*_6_) spectrum of **2**.

Figure **S4**. ^1^H NMR (400 MHz, DMSO-*d*_6_) spectrum of **3**.

Figure **S5**. ^1^H NMR (400 MHz, DMSO-*d*_6_) spectrum of **4**.

Figure **S6**. ^13^C NMR (176 MHz, DMSO-*d*_6_) spectrum of **1**.

Figure **S7**. ^13^C NMR (176 MHz, DMSO-*d*_6_) spectrum of **2**.

Figure **S8**. ^13^C NMR (101 MHz, DMSO-*d*_6_) spectrum of **3**.

Figure **S9**. ^13^C NMR (176 MHz, DMSO-*d*_6_) spectrum of **4**.

Figure **S10**. Positive-ion ESI-mass spectrum of **1**.

Figure **S11**. Positive-ion ESI-mass spectrum of **2**.

Figure **S12**. Positive-ion ESI-mass spectrum of **3**.

Figure **S13**. Positive-ion ESI-mass spectrum of **4**.

Figure **S14**. ^1^H-^1^H COSY (700 MHz, DMSO-*d*_6_) spectrum of **3**.

Figure **S15**. ^1^H-^1^H COSY (700 MHz, DMSO-*d*_6_) spectrum of **3-Al^3+^**.

Figure **S16.**. Absorption intensity of **1-4** versus Al^3+^ concentrations.

Figure **S17**. Benesi-Hildebrand plot of **1**-**4**, assuming 1:1 stoichiometries for association between **1**-**4** and Al^3+^ in 0.3 DMSO/bis-tris solution.

Figure **S18**. The Job’s plot examined between **1**-**4** and Al^3+^ by fluorescence.

**Figure S19**. Normalized fluorescence intensity of **1(a), 2(b), 3(c), 4(d)** (1 μM) with metal ions (Na^+^, K^+^, Ag^+^, Mg^2+^, Ca^2+^, Hg^2+^, Pb^2+^, Cd^2+^, Mn^2+^, Ni^2+^, Co^2+^, Cu^2+^, Zn^2+^, Fe^2+^, Fe^3+^, Cr^3+^, and Al^3+^).

**Figure S20.** Fluorescence photo of **1**, **2** and **4** (1 μM) and **1**, **2** and **4** with metal ions(Na^+^, K^+^, Ag^+^, Mg^2+^, Ca^2+^, Hg^2+^, Pb^2+^, Cd^2+^, Mn^2+^, Ni^2+^, Co^2+^, Cu^2+^, Zn^2+^, Fe^2+^, Fe^3+^, Cr^3+^, and Al^3+^) under the irradiation of an ultraviolet lamp (wavelength 365 ± 50 nm).

**Figure S21.** Theoretical calculated ^[1]^ UV-vis absorption spectrum of **1**-**4**.

**Figure S22.** Electrostatic potential surfaces of **2** visualized in GaussView 5.0 (isovalue = 0.03). The arrow indicates the direction of the dipole moment.

**Figure S23.** Electrostatic potential surfaces of **3** visualized in GaussView 5.0 (isovalue = 0.03). The arrow indicates the direction of the dipole moment.

**Figure S24.** The photo of writing on Al^3+^ test paper of **3** with a paper pen dipped in Al^3+^ solution under the irradiation of a 365 nm UV lamp.

**Table S1**. Performance comparison of recently published sensors.

**Table S2.** Primary orbitals which contribute to the calculated transitions of **1**.

**Table S3.** Primary orbitals which contribute to the calculated transitions of **2**.

**Table S4.** Primary orbitals which contribute to the calculated transitions of **3**.

**Table S5.** Primary orbitals which contribute to the calculated transitions of **4**.

**Table S6**. TD-DFT calculated electronic transition configurations for **1, 2, 3, and 4** along with their corresponding excitation energies and oscillator strengths.

**Table S7.** Primary orbitals which contribute to the calculated transitions of **1-Al** (iso = 0.03).

**Table S8.** Primary orbitals which contribute to the calculated transitions of **2-Al** (iso = 0.03).

**Table S9.** Primary orbitals which contribute to the calculated transitions of **3-Al** (iso = 0.03).

**Table S10.** Primary orbitals which contribute to the calculated transitions of **4-Al** (iso = 0.03).

**Table S11**. TD-DFT calculated electronic transition configurations for **1-Al, 2-Al, 3-Al, and 4-Al** along with their corresponding excitation energies and oscillator strengths.

**References.**


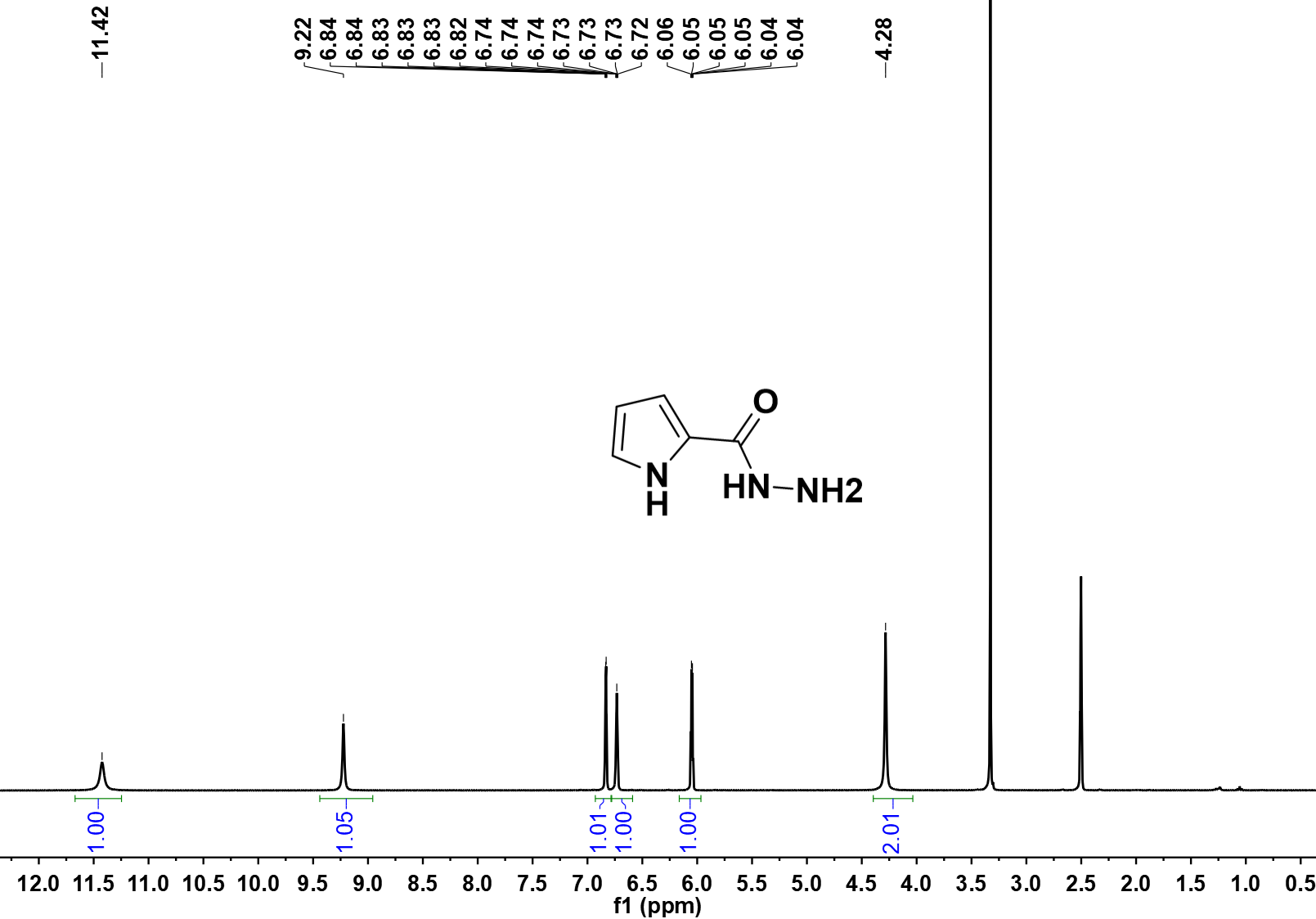


**Figure S1.** ^1^H NMR (400 MHz, DMSO-*d*_6_) spectrum of **1*H*-pyrrole-2-carbohydrazide**.


 **Figure S2.** ^1^H NMR (400 MHz, DMSO-*d*_6_) spectrum of **1**.

**Figure S3.** ^1^H NMR (400 MHz, DMSO-*d*_6_) spectrum of **2.**

**Figure S4.** ^1^H NMR (400 MHz, DMSO-*d*_6_) spectrum of **3**.

**Figure S5.** ^1^H NMR (400 MHz, DMSO-*d*_6_) spectrum of **4**.

**Figure S6.** ^13^C NMR (176 MHz, DMSO-*d*_6_) spectrum of **1**.

**Figure S7.** ^13^C NMR (176 MHz, DMSO-*d*_6_) spectrum of **2**.

**Figure S8.** ^13^C NMR (101 MHz, DMSO-*d*_6_) spectrum of **3**.

**Figure S9.** ^13^C NMR (176 MHz, DMSO-*d*_6_) spectrum of **4**.


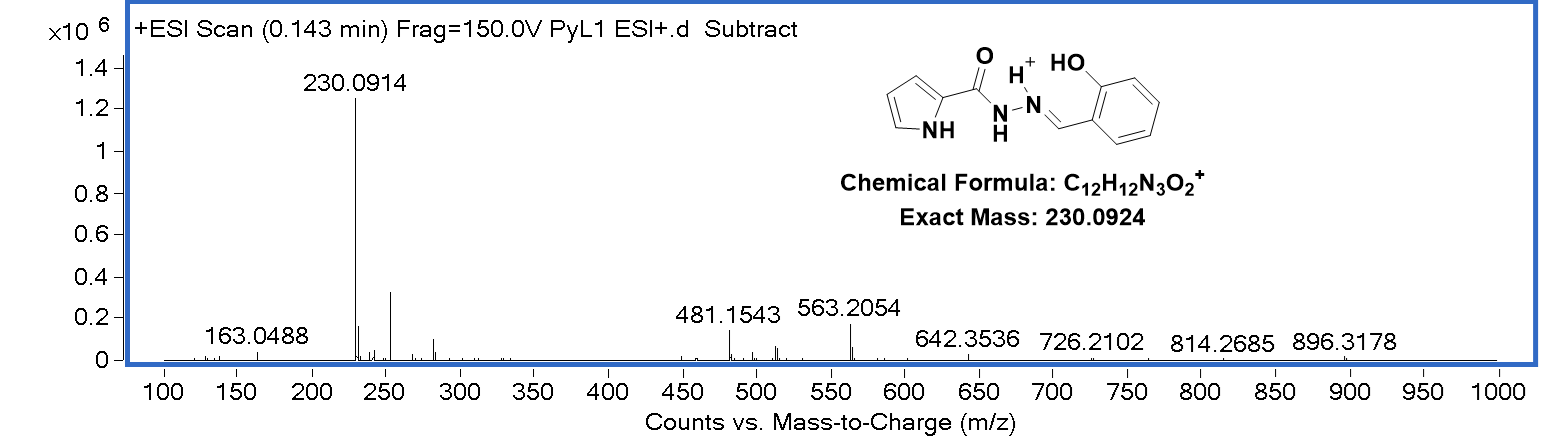


**Figure S10.** Positive-ion ESI-mass spectrum of **1** (100 μM).


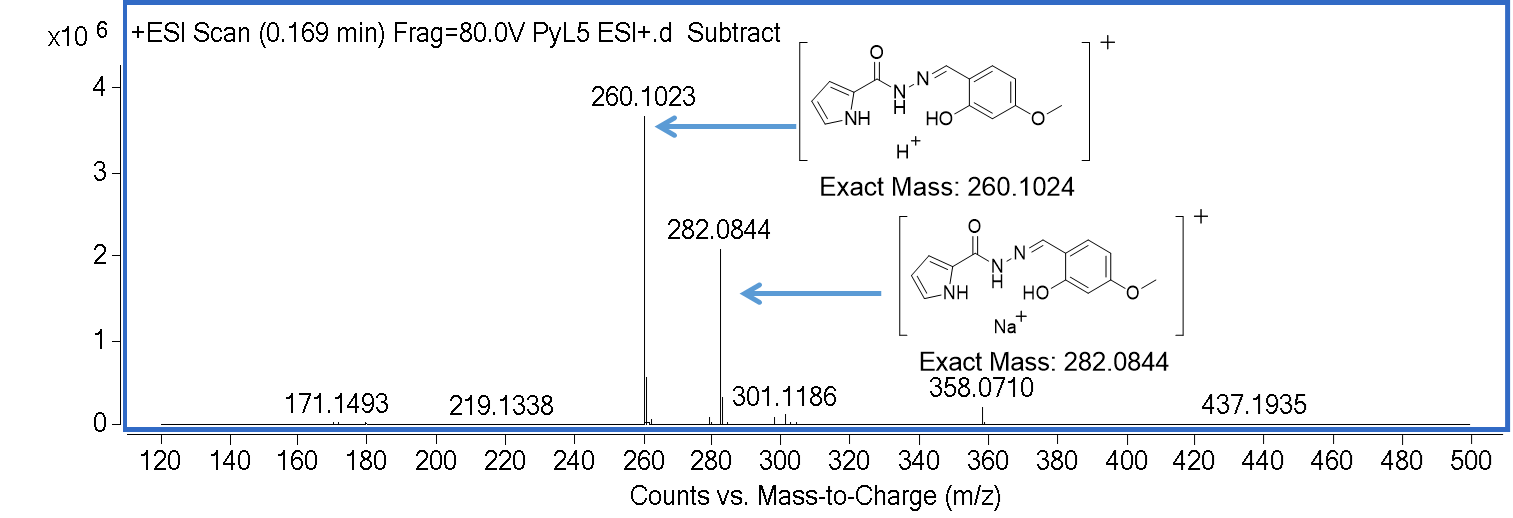


**Figure S11.** Positive-ion ESI-mass spectrum of **2** (100 μM).


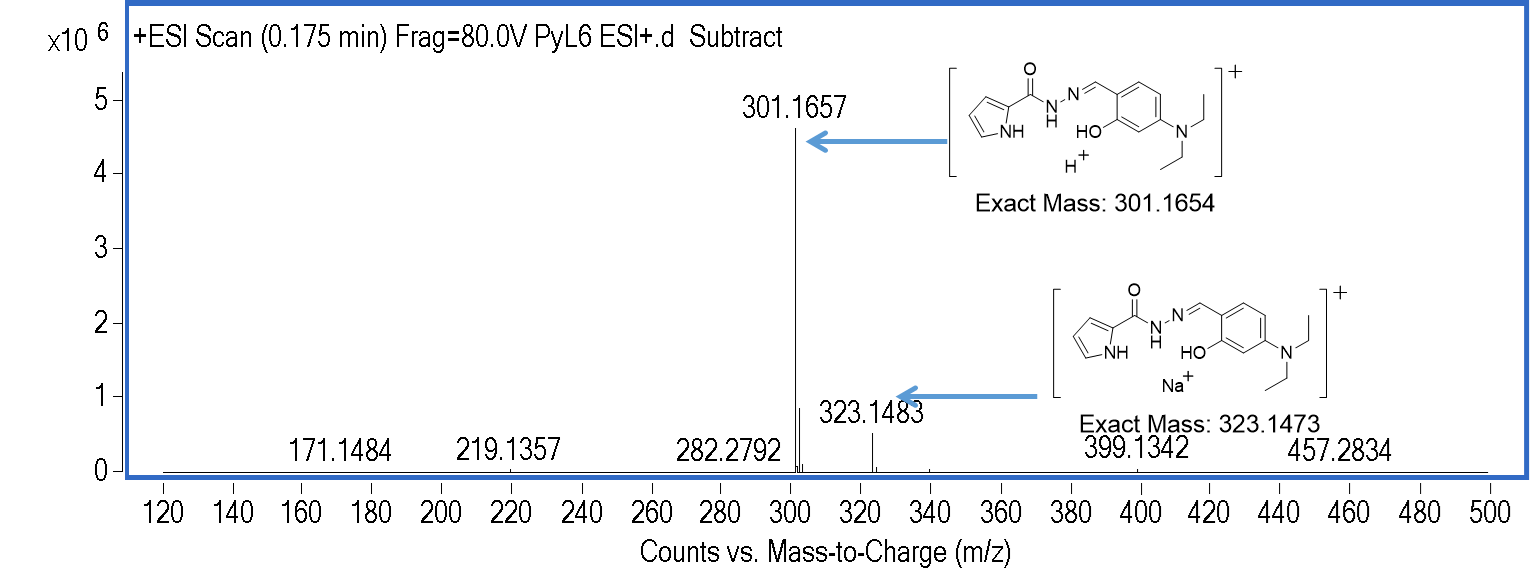


**Figure S12.** Positive-ion ESI-mass spectrum of **3** (100 μM).


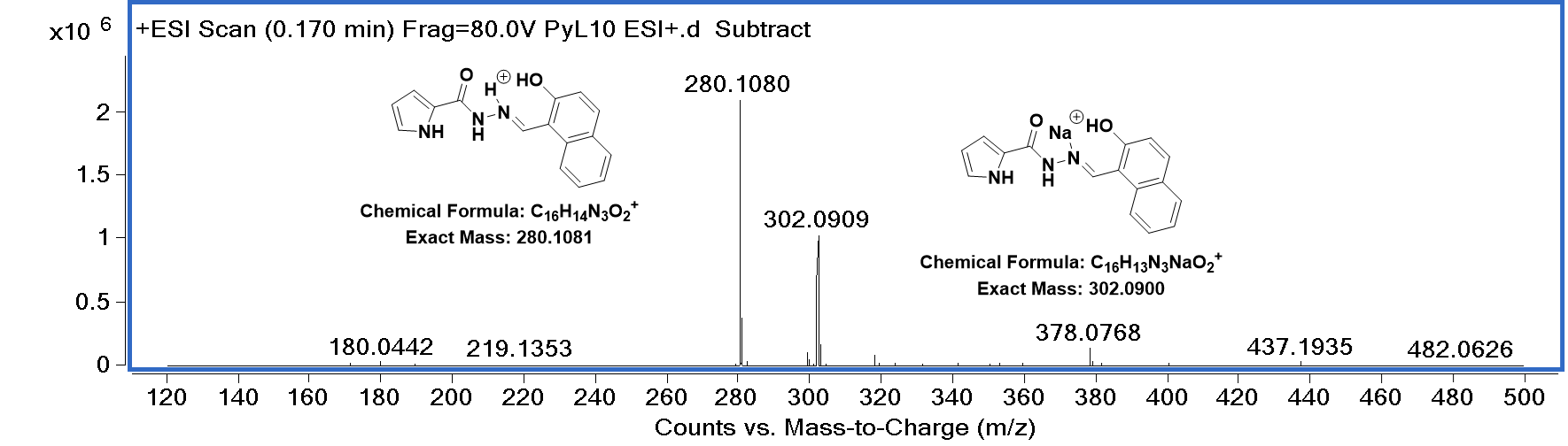


**Figure S13.** Positive-ion ESI-mass spectrum of **4** (100 μM).

**Figure S14.** ^1^H-^1^H COSY (700 MHz, DMSO-*d*_6_) spectrum of **3**.

**Figure S15.** ^1^H-^1^H COSY (700 MHz, DMSO-*d*_6_) spectrum of **3-Al^3+^**.


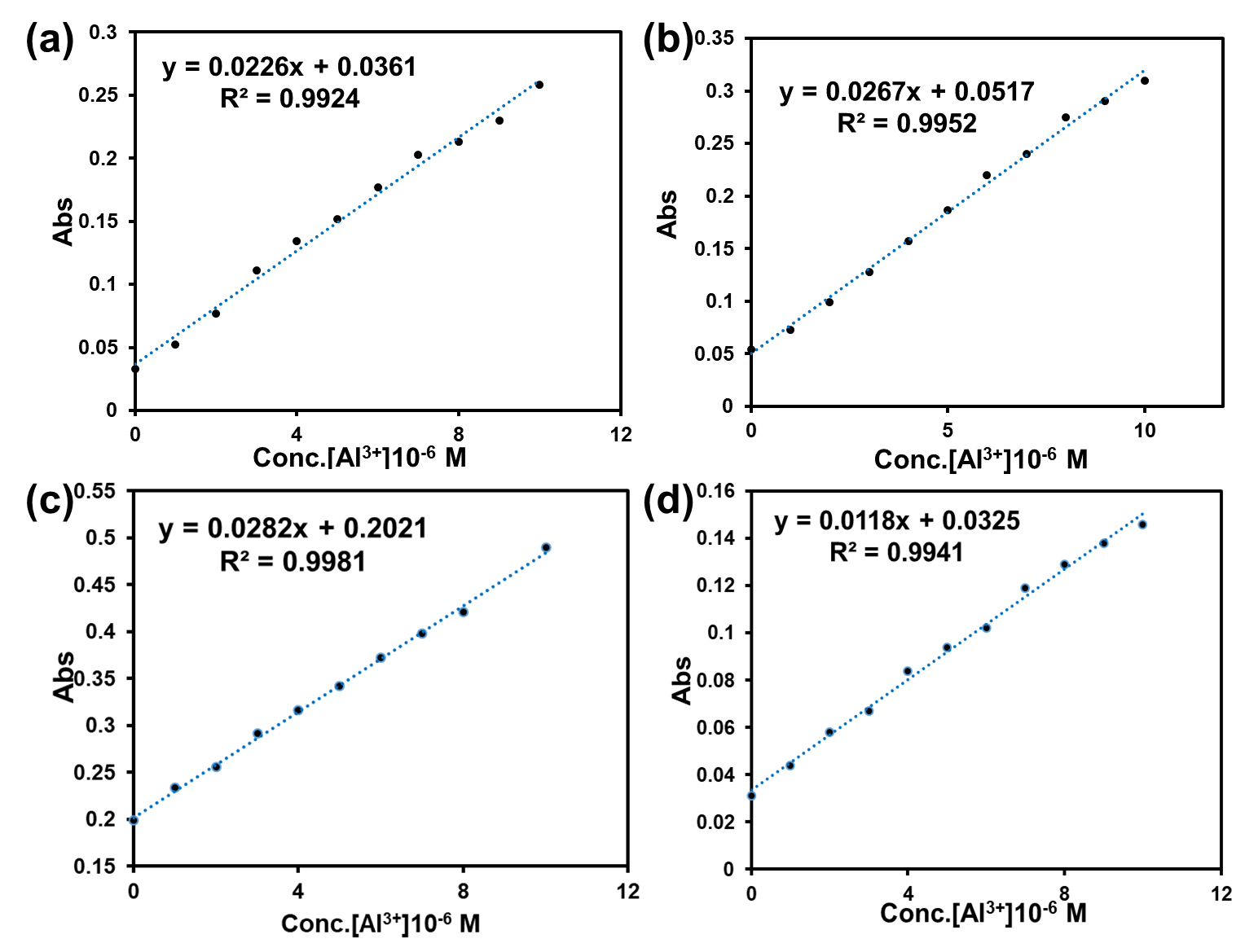


**Figure S16.**. Absorption intensity of **1(a), 2(b), 3(c), 4(d)** (10 μM) versus Al^3+^ concentrations.


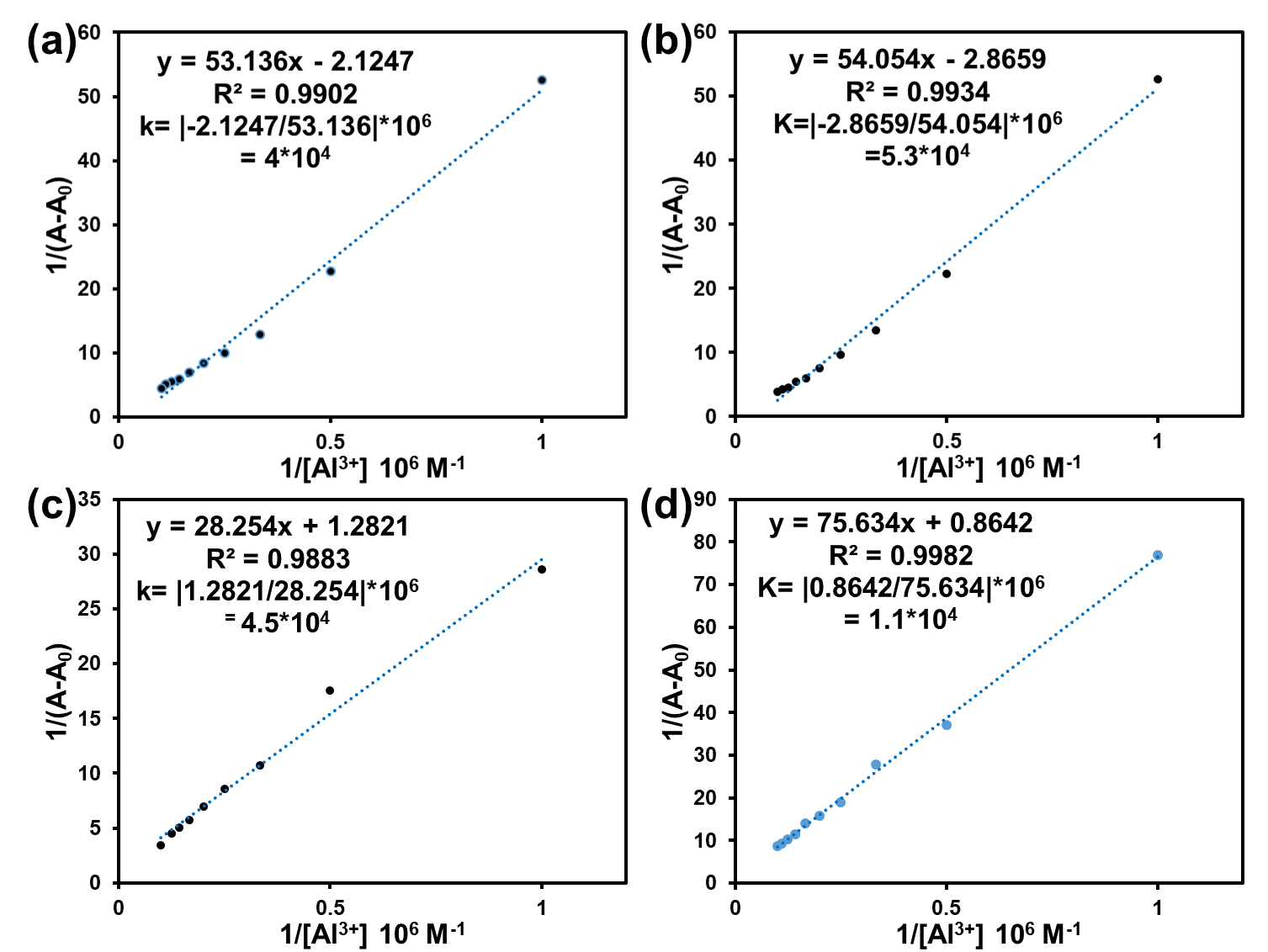


**Figure S17.** Benesi-Hildebrand plot of **1(a), 2(b), 3(c), 4(d)** (10 μM), assuming 1:1 stoichiometries for association between **1**-**4** and Al^3+^ in 0.3 DMSO/bis-tris solution.


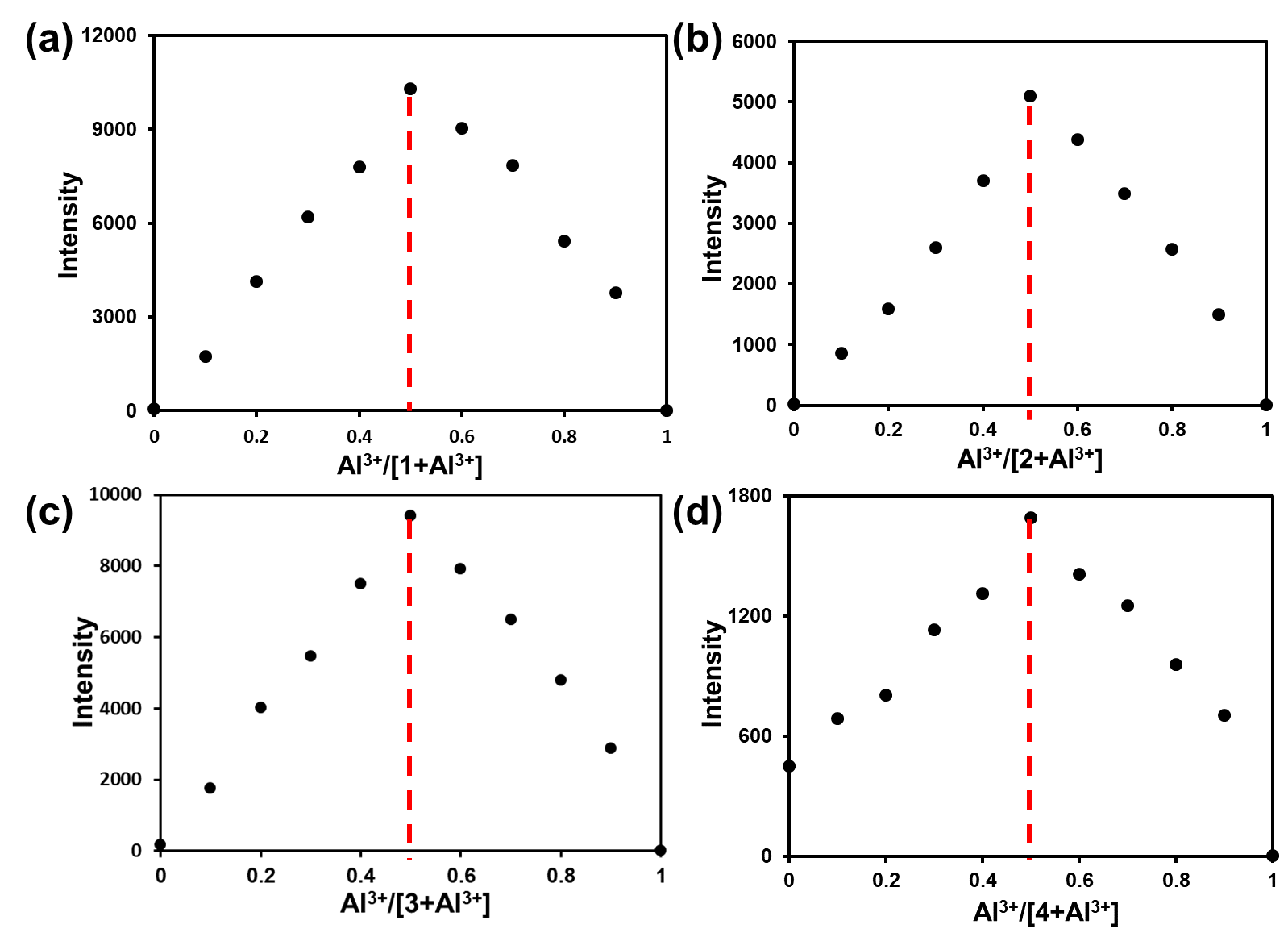


**Figure S18.** The Job’s plot examined between **1(a), 2(b), 3(c), 4(d)** and Al^3+^ by fluorescence.


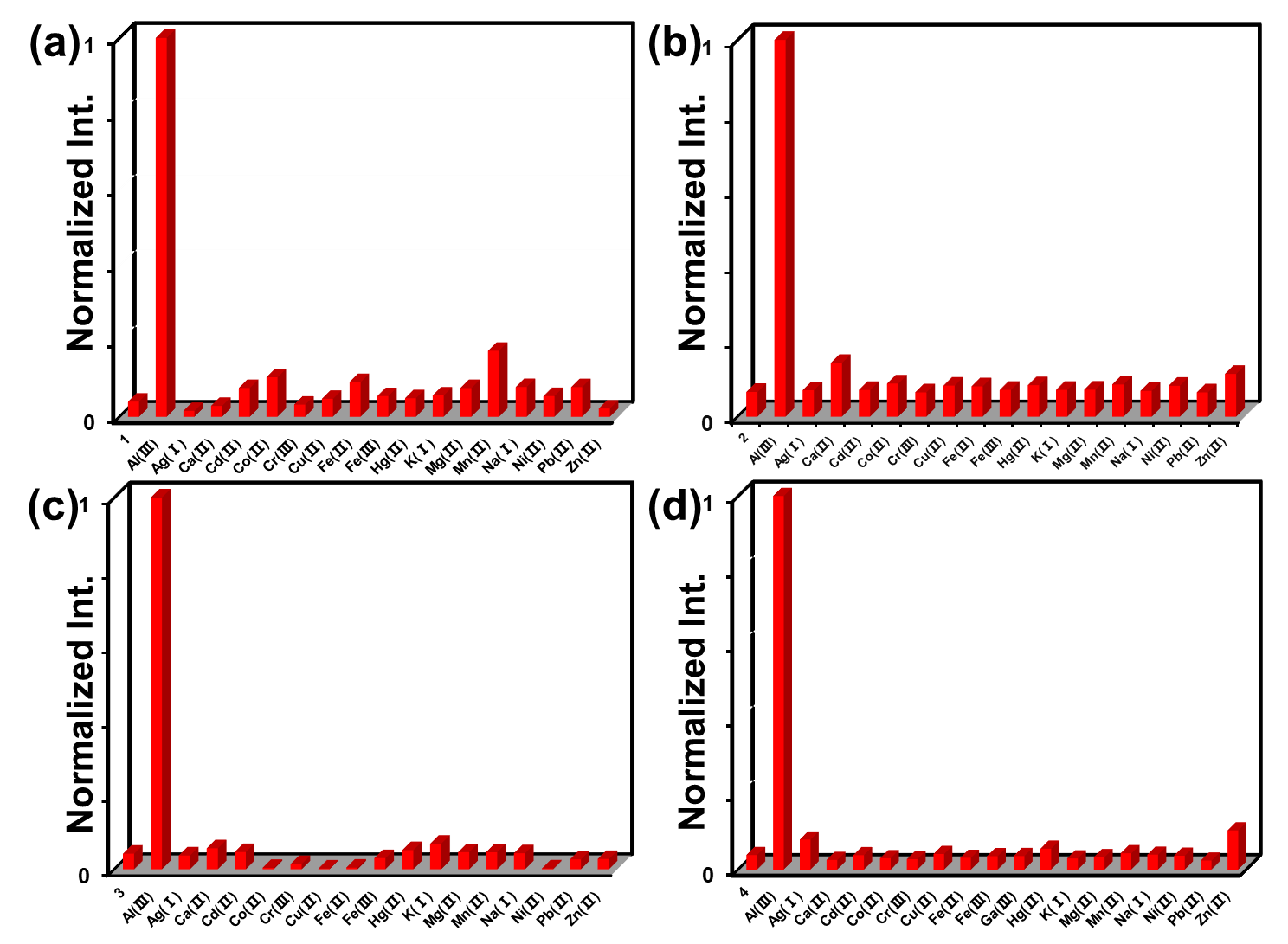


**Figure S19**. Normalized fluorescence intensity of **1(a), 2(b), 3(c), 4(d)** (1 μM) with metal ions (Na^+^, K^+^, Ag^+^, Mg^2+^, Ca^2+^, Hg^2+^, Pb^2+^, Cd^2+^, Mn^2+^, Ni^2+^, Co^2+^, Cu^2+^, Zn^2+^, Fe^2+^, Fe^3+^, Cr^3+^, and Al^3+^).


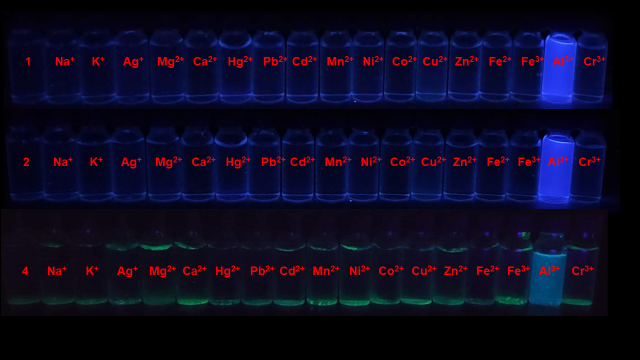


**Figure S20.** Fluorescence photo of **1**, **2** and **4** (1 μM) and **1**, **2** and **4** with metal ions(Na^+^, K^+^, Ag^+^, Mg^2+^, Ca^2+^, Hg^2+^, Pb^2+^, Cd^2+^, Mn^2+^, Ni^2+^, Co^2+^, Cu^2+^, Zn^2+^, Fe^2+^, Fe^3+^, Cr^3+^, and Al^3+^) under the irradiation of an ultraviolet lamp (wavelength 365 ± 50 nm).

**Figure S21.** Theoretical calculated ^[1]^ UV-vis absorption spectrum of **1**-**4**.


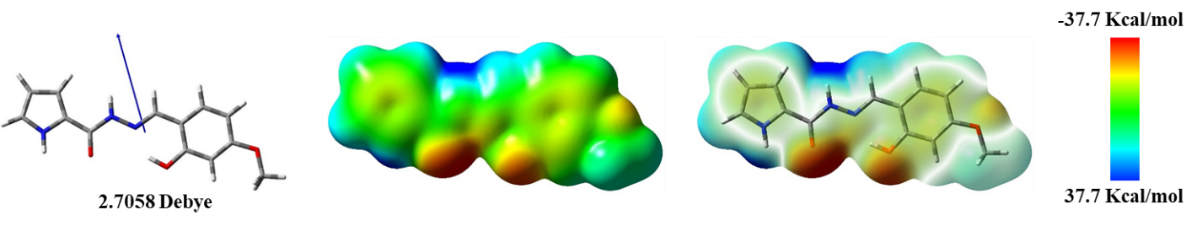


**Figure S22.** Electrostatic potential surfaces of **2** visualized in GaussView 5.0 (isovalue = 0.03). The arrow indicates the direction of the dipole moment.


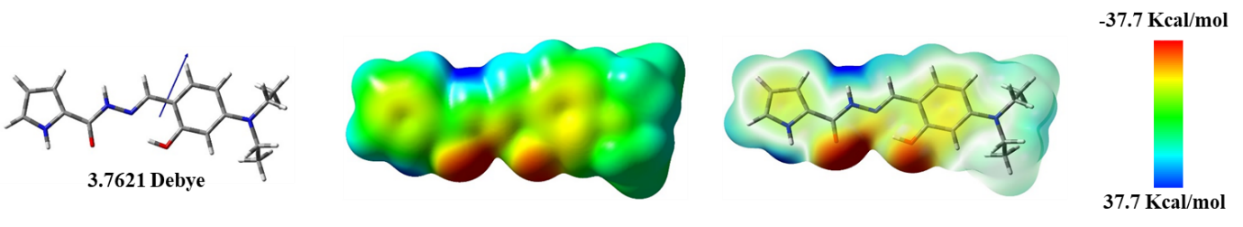


**Figure S23.** Electrostatic potential surfaces of **3** visualized in GaussView 5.0 (isovalue = 0.03). The arrow indicates the direction of the dipole moment.


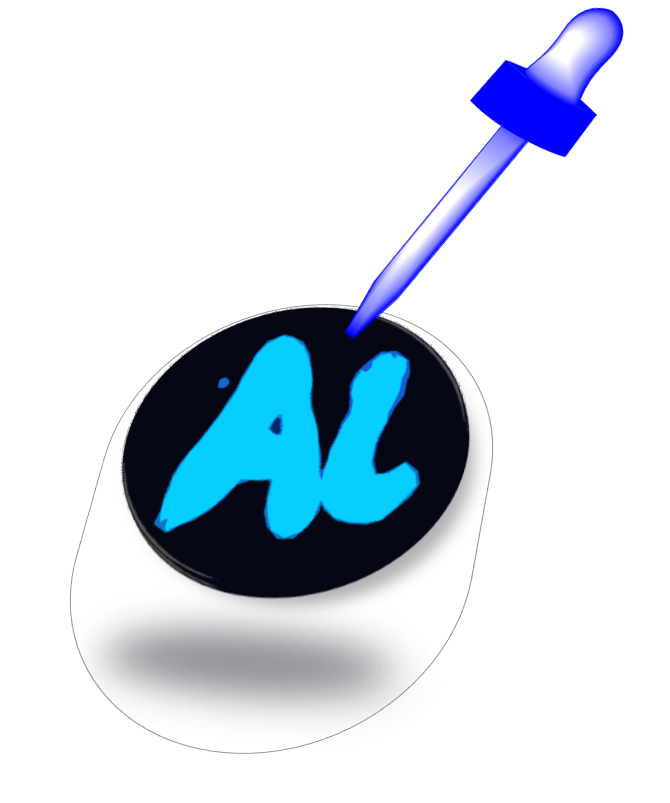


**Figure S24.** The photo of writing on Al^3+^ test paper of **3** with a paper pen dipped in Al^3+^ solution under the irradiation of a 365 nm UV lamp.

**Table S1**. Performance comparison of recently published sensors.

| Entry | Sensors | Media | Tagart | LOD(M) | Ka(M^-1^) | Reference |
| --- | --- | --- | --- | --- | --- | --- |
| 1 |  | EtOH | Al^3+^ | 1×10^-7^ | 2.5×10^3^ | [1] |
| 2 |  | MeOH/  PBS | Al^3+^ | 3.1×10^-7^ | 2.61×10^5^ | [2] |
| 3 |  | MeCN/  H_2_O | Al^3+^ | 1.08×10^-6^ | 2.1×10^2^ | [3] |
| 4 |  | DMSO/  H_2_O | Al^3+^ | 1.48×10^-8^  4.23×10^-8^ | 9.4×10^4^  9.4×10^4^ | [4] |
| 5 |  | H_2_O | Al^3+^ | 6.2×10^-8^ | 1.89×10^4^ | [5] |
| 6 |  | DMF | Al^3+^ | 3.9×10^-6^ | 2×10^7^ | [6] |
| 7 |  | EtOH/  H_2_O | Al^3+^ | 5.78×10^-8^ | 5.74×10^9^ | [7] |
| 8 |  | H_2_O | Al^3+^ | 5.3×10^-8^  4.5×10^-8^  4.2×10^-8^  1.02×10^-7^ | 4.0 × 10^4^  5.3 × 10^4^  4.5 × 10^4^  1.1 × 10^4^ | This  work |

**Table S2.** Primary orbitals which contribute to the calculated transitions of **1** (iso = 0.03). ^[8]^


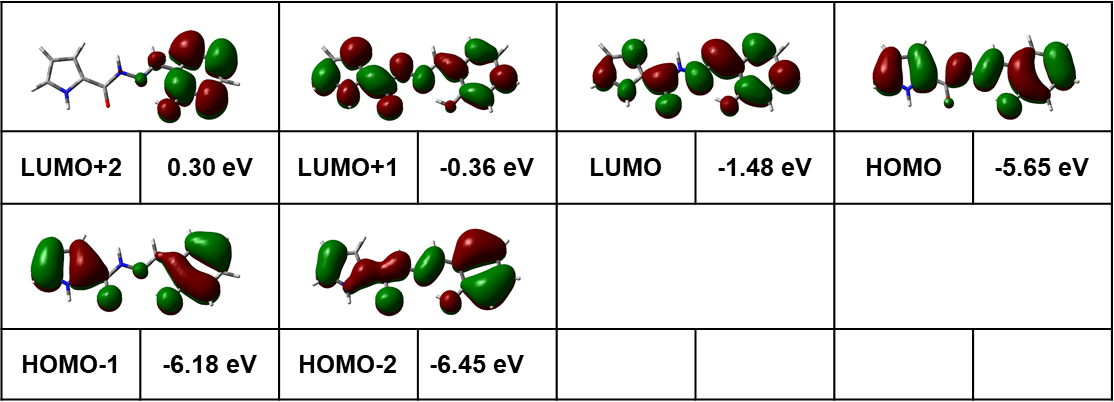


**Table S3.** Primary orbitals which contribute to the calculated transitions of **2** (iso = 0.03).


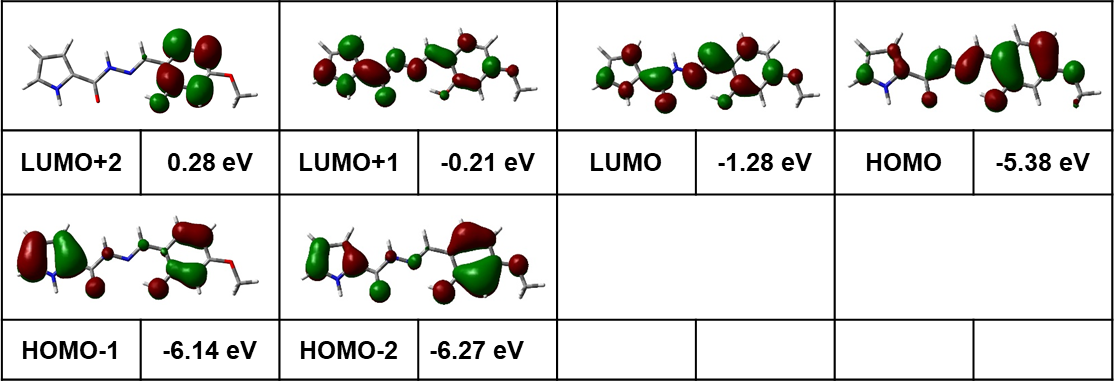


**Table S4.** Primary orbitals which contribute to the calculated transitions of **3** (iso = 0.03).


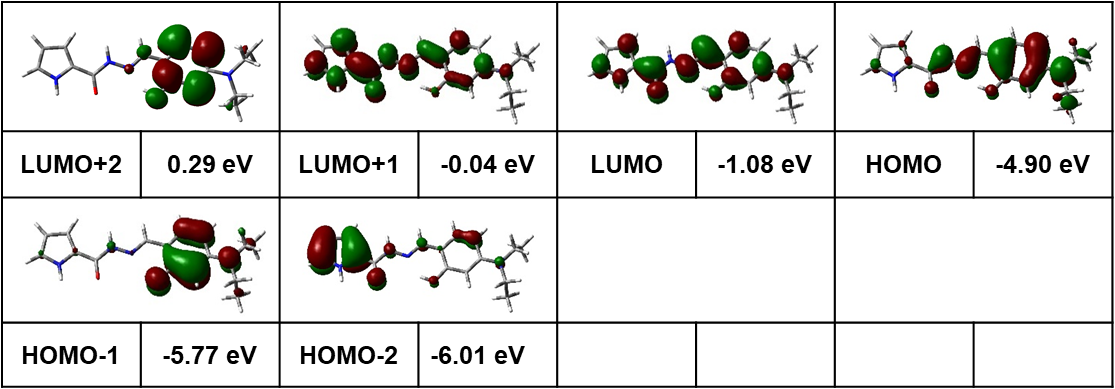


**Table S5.** Primary orbitals which contribute to the calculated transitions of **4** (iso = 0.03).


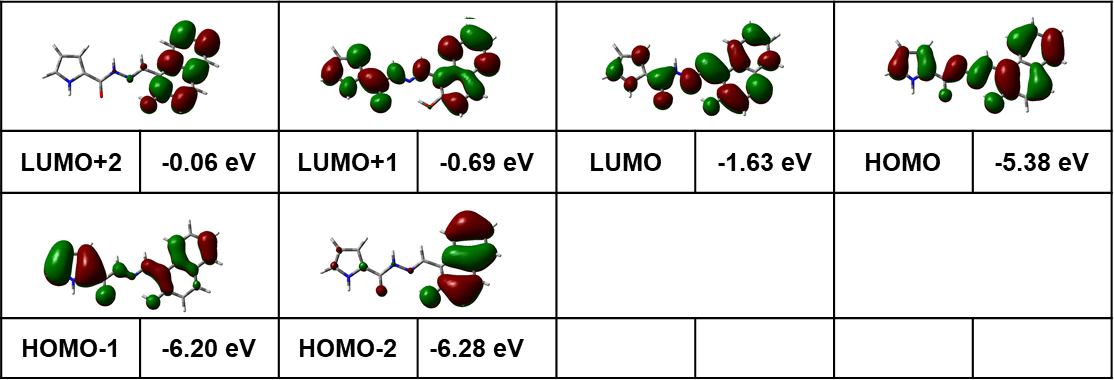


**Table S6**. TD-DFT calculated electronic transition configurations for **1, 2, 3, and 4** along with their corresponding excitation energies and oscillator strengths.


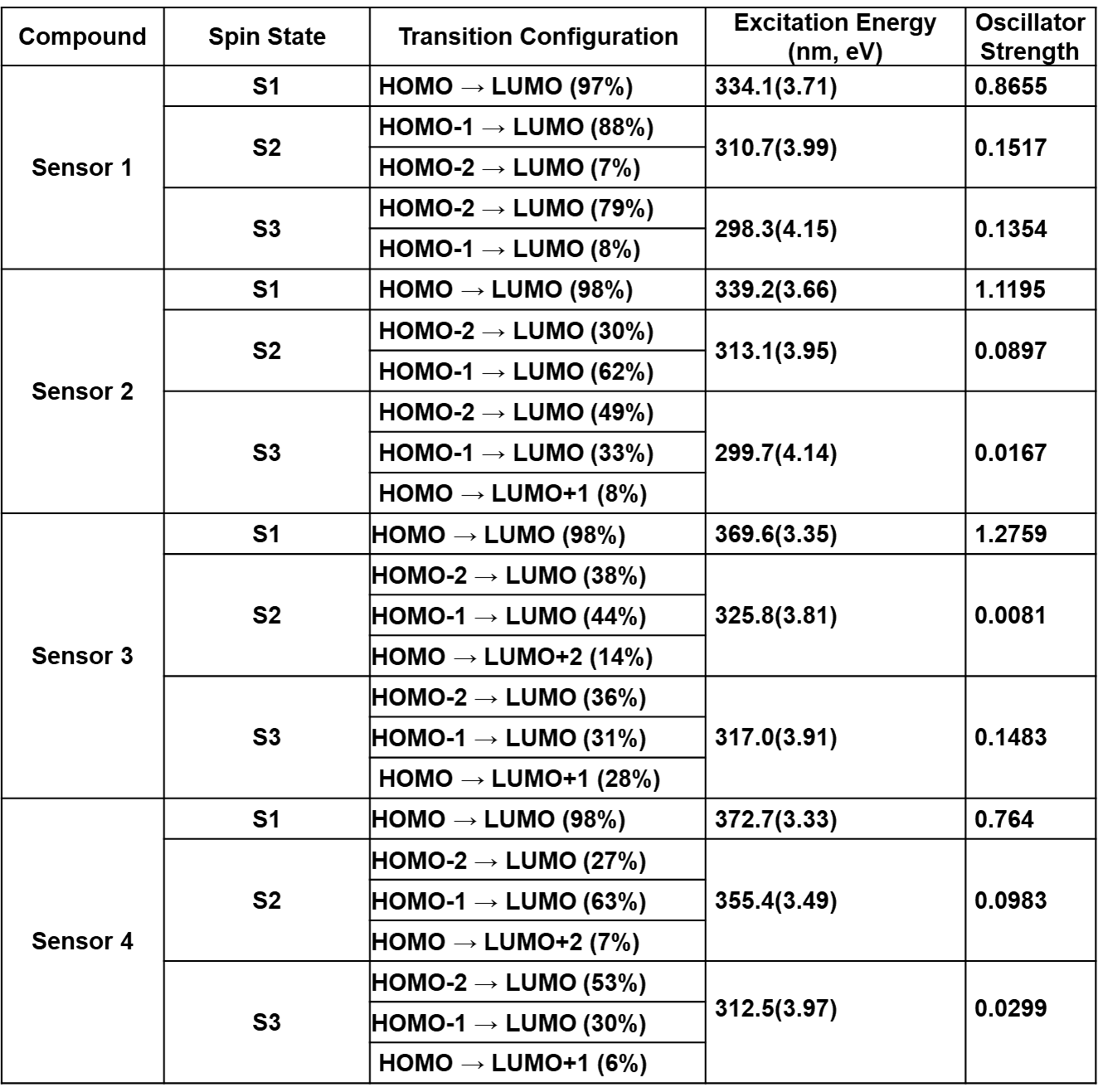


**Table S7.** Primary orbitals which contribute to the calculated transitions of **1-Al** (iso = 0.03).


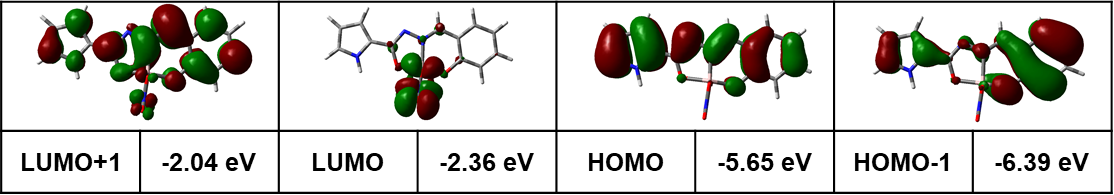


**Table S8.** Primary orbitals which contribute to the calculated transitions of **2-Al** (iso = 0.03).


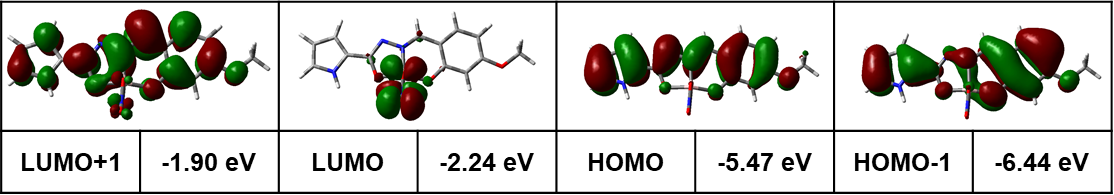


**Table S9.** Primary orbitals which contribute to the calculated transitions of **3-Al** (iso = 0.03).


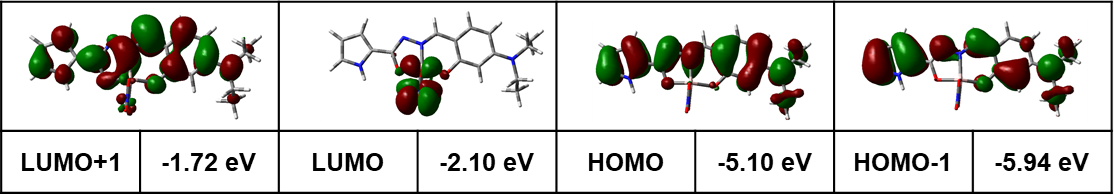


**Table S10.** Primary orbitals which contribute to the calculated transitions of **4-Al** (iso = 0.03).


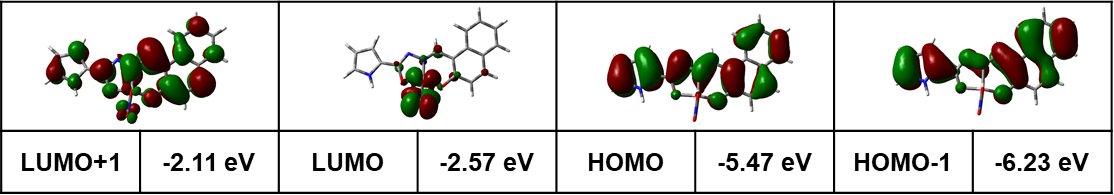


**Table S11**. TD-DFT calculated electronic transition configurations for **1+Al, 2+Al, 3+Al, and 4+Al** along with their corresponding excitation energies and oscillator strengths.


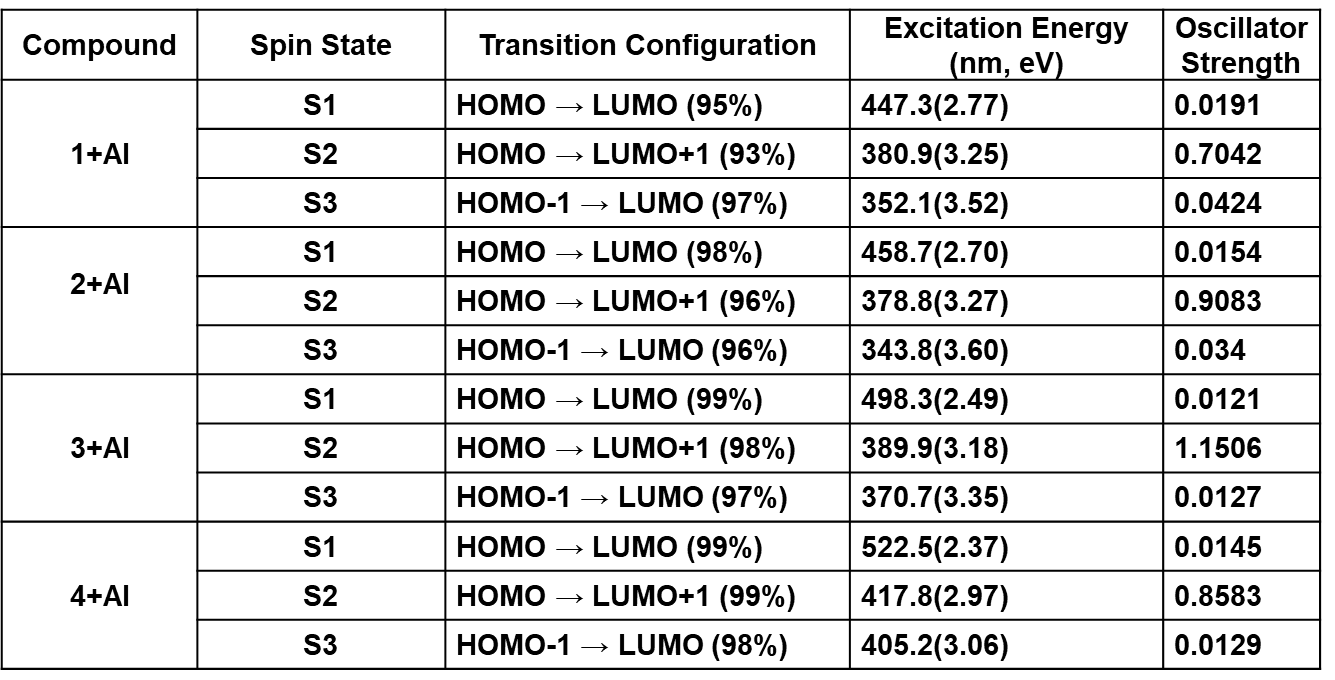


**References:**

[1] L. Fan, J.C. Qin, C.R. Li and Z.Y. Yang, *Spectrochim. Acta*, 2019, **218**, 342-347.

[2] H.Y. Liu, B.B. Zhang, C.Y. Tan, F. Liu, J.K. Cao, Y. Tan and Y.Y. Jiang, *Talanta*, 2016, **161**, 309-319.

[3] T. Anand, G. Sivaraman, A. Mahesh and D. Chellappa, *Anal. Chim. Acta*, 2015, **853**, 596-601

[4] J. Fu, B. Li, H. Mei, Y. Chang and K. Xu, *Spectrochimica Acta Part A: Molecular and Biomolecular Spectroscopy*, 2020, **227**, 117678

[5] Y. Wang, Y.F. Song, L. Zhang, G.G. Dai, R.F. Kang, W. N. Wu, Z.H. Xu and Y.C. Fan, L.Y. Bian, *Talanta,* 2019, **203**, 178–185.

[6] Y.W. Choi, G.J. Park, Y.J. Na, H.Y. Jo, S.A. Lee, G.R. You and C. Kim, *Sensor Actuator B Chem.*, 2014, **194**, 343–352.

[7] D.P. Singh, R. Dwivedi, A.K. Singh, B. Koch, P. Singh and V.P. Singh, *Sensor Actuator B Chem.*, 2017, **238**, 128–137.

[8] M. J. Frisch, G. W. Trucks, H. B. Schlegel, G. E. Scuseria, M. A. Robb, J. R. Cheeseman, G. Scalmani, V. Barone, B. Mennucci, G. A. Petersson, H. Nakatsuji, M. Caricato, X. Li, H. P. Hratchian, A. F. Izmaylov, J. Bloino, G. Zheng, J. L. Sonnenberg, M. Hada, M. Ehara, K. Toyota, R. Fukuda, J. Hasegawa, M. Ishida, T. Nakajima, Y. Honda, O. Kitao, H. Nakai, T. Vreven, J. A. Montgomery, Jr., J. E. Peralta, F. Ogliaro, M. Bearpark, J. J. Heyd, E. Brothers, K. N. Kudin, V. N. Staroverov, R. Kobayashi, J. Normand, K. Raghavachari, A. Rendell, J. C. Burant, S. S. Iyengar, J. Tomasi, M. Cossi, N. Rega, J. M. Millam, M. Klene, J. E. Knox, J. B. Cross, V. Bakken, C. Adamo, J. Jaramillo, R. Gomperts, R. E. Stratmann, O. Yazyev, A. J. Austin, R. Cammi, C. Pomelli, J. W. Ochterski, R. L. Martin, K. Morokuma, V. G. Zakrzewski, G. A. Voth, P. Salvador, J. J. Dannenberg, S. Dapprich, A. D. Daniels, O. Farkas, J. B. Foresman, J. V. Ortiz, J. Cioslowski, and D. J. Fox, Gaussian, Inc., Wallingford CT, 2009.

# Supplementary Data

Supplementary Material should be uploaded separately on submission. Please include any supplementary data, figures and/or tables. All supplementary files are deposited to FigShare for permanent storage and receive a DOI.

Supplementary material is not typeset so please ensure that all information is clearly presented, the appropriate caption is included in the file and not in the manuscript, and that the style conforms to the rest of the article. To avoid discrepancies between the published article and the supplementary material, please do not add the title, author list, affiliations or correspondence in the supplementary files.

# Supplementary Figures and Tables

For more information on Supplementary Material and for details on the different file types accepted, please see [here](http://home.frontiersin.org/about/author-guidelines#SupplementaryMaterial). Figures, tables, and images will be published under a Creative Commons CC-BY licence and permission must be obtained for use of copyrighted material from other sources (including re-published/adapted/modified/partial figures and images from the internet). It is the responsibility of the authors to acquire the licenses, to follow any citation instructions requested by third-party rights holders, and cover any supplementary charges.

## Supplementary Figures

**
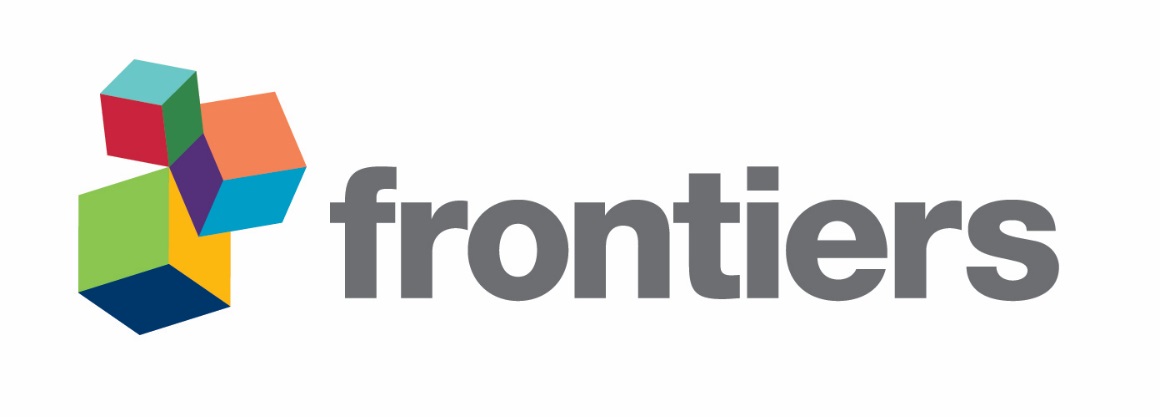
**

**Supplementary Figure 1.** The figure legends are required to have the same font as the main text, 12 point normal Times New Roman, single spaced. Please use a single paragraph for each legend and prepare the figures keeping in mind the PDF layout.
